# IL-33 promotes anemia during chronic inflammation by inhibiting differentiation of erythroid progenitors

**DOI:** 10.1084/jem.20200164

**Published:** 2020-06-10

**Authors:** James W. Swann, Lada A. Koneva, Daniel Regan-Komito, Stephen N. Sansom, Fiona Powrie, Thibault Griseri

**Affiliations:** Kennedy Institute of Rheumatology, University of Oxford, Oxford, UK

## Abstract

An important comorbidity of chronic inflammation is anemia, which may be related to dysregulated activity of hematopoietic stem and progenitor cells (HSPCs) in the bone marrow (BM). Among HSPCs, we found that the receptor for IL-33, ST2, is expressed preferentially and highly on erythroid progenitors. Induction of inflammatory spondyloarthritis in mice increased IL-33 in BM plasma, and IL-33 was required for inflammation-dependent suppression of erythropoiesis in BM. Conversely, administration of IL-33 in healthy mice suppressed erythropoiesis, decreased hemoglobin expression, and caused anemia. Using purified erythroid progenitors in vitro, we show that IL-33 directly inhibited terminal maturation. This effect was dependent on NF-κB activation and associated with altered signaling events downstream of the erythropoietin receptor. Accordingly, IL-33 also suppressed erythropoietin-accelerated erythropoiesis in vivo. These results reveal a role for IL-33 in pathogenesis of anemia during inflammatory disease and define a new target for its treatment.

## Teaser

Erythroid progenitors preferentially express ST2, the receptor for IL-33; this cytokine is necessary and sufficient to cause anemia during chronic inflammation. IL-33 directly inhibits differentiation of erythroid progenitors by activating NF-κB signaling, which is normally downregulated in erythropoiesis.

## Introduction

Chronic inflammatory diseases cause local effects in diseased organs, but owing to the systemic effects of inflammation they are frequently associated with important comorbidities in other locations, including the blood and bone marrow (BM; Argollo et al., 2019; Turesson, 2016). This principle is exemplified by patients with rheumatoid arthritis and spondyloarthritis (SpA), a complex of diseases causing chronic inflammation of the axial spine, peripheral joints, and small intestine (Ranganathan et al., 2017), who may also develop anemia of inflammatory disease (AID) or BM dysfunction (Furst et al., 2013; Smith et al., 1992).

AID is an important cause of morbidity in people, limiting physical activity and impairing quality of life in ∼1 million adults in the United States (Nemeth and Ganz, 2014). Its pathogenesis is multifactorial, but important contributors include reduced absorption of iron, decreased secretion of or responsiveness to erythropoietin (EPO), and suppressive effects of inflammatory cytokines on erythroid progenitors in BM (Nemeth and Ganz, 2014; Weiss and Goodnough, 2005).

Contrary to their traditional image as isolated cells in secluded niches, hematopoietic stem and progenitor cells (HSPCs), including erythroid progenitors, express receptors for inflammatory cytokines, rendering them highly responsive to signals emanating from inflamed sites during disease (King and Goodell, 2011; Takizawa et al., 2012). Much attention has focused on the lineage-instructive effects of these cytokines on multipotent HSPCs, with both IL-1β (Pietras et al., 2016) and TNF-α (Etzrodt et al., 2019) causing early adoption of myeloid transcriptional signatures in primitive stem cells. However, cytokines may also deliver permissive or inhibitory signals to progenitors that have already committed to one lineage, modulating hematopoietic output to meet demand. In previous studies, IL-6 (McCranor et al., 2014), TNF-α (Xiao et al., 2002), and IFN-γ (Felli et al., 2005; Libregts et al., 2011) have exerted suppressive effects on erythropoiesis in various settings, but the relative importance of these and other as-yet-unknown factors in causing AID, and the progenitor stages on which they act, remain unclear.

The pathogenesis of SpA, which has been elucidated partly by studies of the murine SKG model (Benham et al., 2014; Ruutu et al., 2012), depends on contributions from the innate and adaptive immune systems, coordinated by a network of proinflammatory cytokines, including TNF-α, IL-6, IL-17, and IL-23 (Benham et al., 2014; Hata et al., 2004). To understand the lineage-specific effects and importance of these and other cytokines in the development of AID during SpA, we compared expression of cytokine receptors between myeloid and erythroid progenitors, finding that the receptor for IL-33 is expressed preferentially among the latter. IL-33 is a member of the IL-1 family of cytokines released from fibroblasts and endothelial and epithelial cells at barrier surfaces in response to damage (Liew et al., 2016). Its receptor is composed of two components: the specific chain ST2 and the IL-1 receptor accessory protein, shared with other IL-1 family members. IL-33 activates several types of ST2^+^ mature hematopoietic cell but, aside from a role in eosinopoiesis (Johnston et al., 2016), its impact on HSPCs has received little attention.

In this study, we find that IL-33 has an important and novel role in suppressing erythropoiesis in a mouse model of chronic inflammatory SpA and could recreate features of AID when injected in vivo. We demonstrate that IL-33 exerts a direct effect on erythroid progenitors in vitro to inhibit differentiation into mature RBCs, contingent on activation of NF-κB, and inhibits EPO-induced signaling pathways downstream of the EPO receptor (EPO-R) in a mechanism unrelated to those of previously described suppressors of erythropoiesis.

## Results

### SpA perturbs hematopoiesis and causes AID

**Figure 1.**
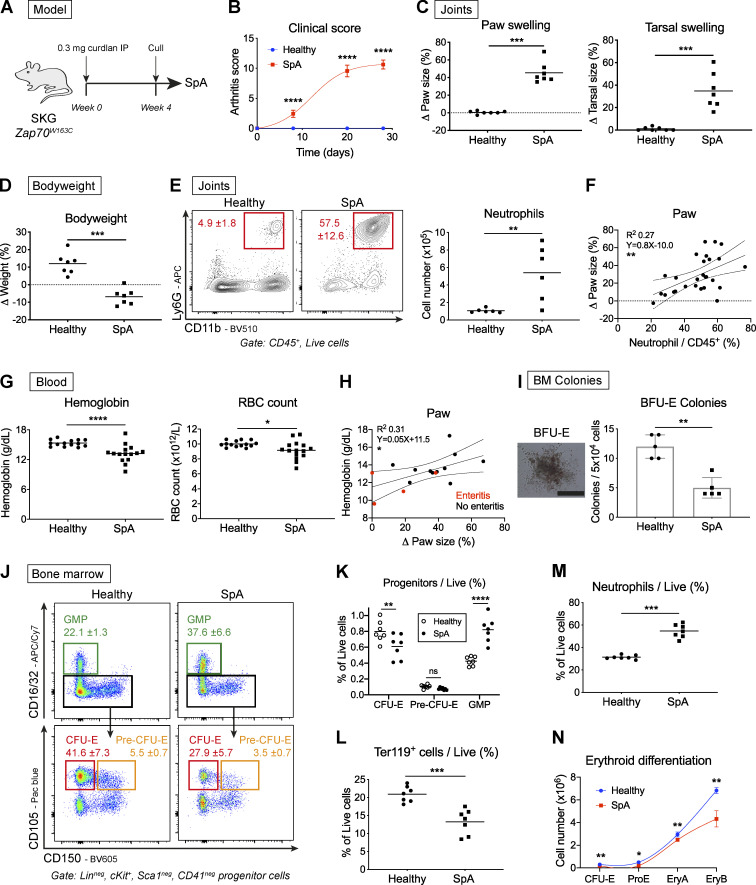
**SpA in mice causes dysregulated hematopoiesis characterized by enhanced myelopoiesis and suppressed erythropoiesis.** **(A)** SpA was induced in SKG mice by injecting curdlan IP. Mice developed inflammatory arthritis and were culled after 4 wk, yielding data shown in B–N. Littermate “healthy” control mice were injected with PBS and culled after 4 wk. **(B)** Clinical arthritis score (mean and SD, *n* = six per group, two-way ANOVA with Sidak’s multiple comparison test, representative of four independent experiments). **(C)** Changes in swelling of paws and tarsi (ankles) averaged for each mouse (points show individual mice with mean, Mann-Whitney *U* test, representative of four independent experiments). **(D)** Changes in bodyweight (points show individual mice with mean, Mann-Whitney *U* test, representative of four independent experiments). **(E)** Representative images and frequency of neutrophils (CD11b^+^Ly6G^+^) among CD45^+^ cells infiltrating joints (mean and SD, *n* = six per group, Mann-Whitney *U* test, representative of four independent experiments). **(F)** Scatterplot of paw swelling against neutrophil infiltration (points show individual mice with linear regression line and 95% confidence intervals, values pooled from three independent experiments). **(G)** Blood Hgb and RBC concentrations (points show individual mice with mean, Mann-Whitney *U* test, representative of two independent experiments). **(H)** Scatterplot of blood Hgb concentration against paw swelling. Red dots show mice with gross enteritis (points show individual mice with linear regression line and 95% confidence intervals, values pooled from three independent experiments). **(I)** Representative image and frequencies of BFU-E colonies from whole BM after 10 d (scale bar = 500 µm, points show individual mice with mean and SD, Mann-Whitney *U* test, representative of two independent experiments). **(J)** Representative flow cytometric images of BM progenitor populations (mean and SD for proportion of cells among lineage (Lin)^neg^, cKit^+^, Sca1^neg^, and CD41^neg^ progenitors, *n* = seven mice per group, representative of four independent experiments). **(K)** Proportion of indicated BM progenitors among live BM cells (points show individual mice with mean, two-way ANOVA with Sidak’s multiple comparison test, representative of four independent experiments). **(L and M)** Frequencies of mature erythroid cells (L; Ter119^+^) and neutrophils (M; CD11b^+^Ly6G^+^) among live BM cells (points show individual mice with mean, Mann-Whitney *U* test, representative of four independent experiments). **(N)** Erythroid differentiation pathway (mean and SD for *n* = five mice per group, lines show locally weighted scatterplot smoothing [LOWESS] spline, Mann-Whitney *U* test, representative of four independent experiments). EryA (cKit^int^, CD71^+^, Ter119^int^), erythroblast A (CD71^+^, Ter119^+^, FSC^hi^); Ery B, erythroblast B (CD71^int^, Ter119^+^, FSC^int^); ProE, proerythroblast. *, P < 0.05; **, P < 0.01; ***, P < 0.001; ****, P < 0.0001; ns, not significant.

**Figure S1.**
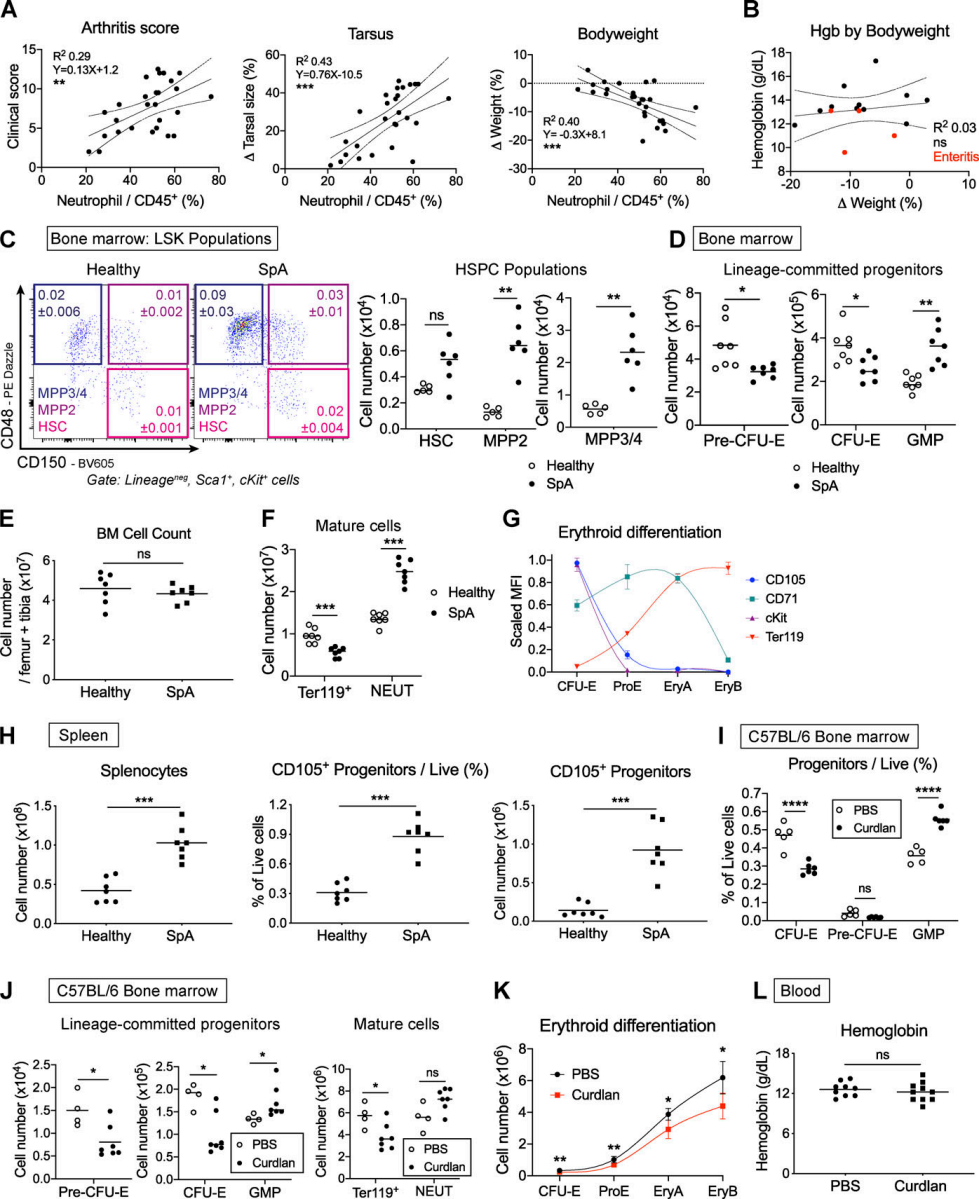
**Curdlan injection perturbs hematopoiesis in SKG and WT C57BL/6 mice.** Data for A–H are from SKG mice injected with curdlan to induce SpA or littermate controls injected with PBS. **(A)** Scatterplots of clinical score (left), change in tarsal size (middle), and bodyweight (right) against neutrophil infiltration of the paws. Points represent individual mice, and solid lines represent linear regression model with 95% confidence intervals (dotted lines); values were pooled from three independent experiments. **(B)** Dot plot of blood Hgb concentration against change in weight. Red dots indicate mice with gross small intestinal inflammation (enteritis). Points represent individual mice, and solid line represents linear regression model with 95% confidence intervals (dotted lines); values were pooled from two independent experiments. **(C)** Representative flow cytometric images of lineage^neg^, Sca1^+^, cKit^+^ (LSK) stem and progenitor cells. HSCs: LSK, CD150^+^, CD48^neg^; MPP2: LSK, CD150^+^, CD48^+^; MPP3/4: LSK, CD150^neg^, CD48^+^. Figures show mean and SD of indicated populations among live BM cells, *n* = five to six per group. Graphs show absolute numbers of indicated cells derived from one femur and one tibia in SpA or healthy mice. Points represent individual mice with mean, Mann-Whitney *U* test, representative of four independent experiments. **(D)** Absolute numbers of BM progenitors derived from one femur and one tibia in SpA or healthy mice (points represent individual mice with mean, Mann-Whitney *U* test, representative of four independent experiments). **(E)** Absolute number of live BM cells derived from one femur and one tibia in indicated groups (points show individual mice with mean, Mann-Whitney *U* test, representative of four independent experiments). **(F)** Absolute number of mature (Ter119^+^) erythroid cells and neutrophils (NEUT) in indicated groups (points show individual mice with mean, Mann-Whitney *U* test, representative of four independent experiments). **(G)** Graph showing expression of surface markers in erythroid progenitor stages used to generate erythroid differentiation pathway graphs. Points represent mean and SD of scaled median fluorescence intensity values for *n* = five mice. Connecting line is locally weighted scatterplot smoothing (LOWESS) spline, representative of four independent experiments. **(H)** Absolute number of live cells from the spleen (left), frequency of erythroid progenitors (lineage^neg^, Sca1^neg^, cKit^+^, CD41^neg^, CD16/32^neg^, CD105^+^) among live cells (middle) and absolute number of erythroid progenitors per spleen (right) in indicated mice (points show individual mice with mean, Mann-Whitney *U* test, representative of four independent experiments). **(I)** C57BL/6 WT littermate mice were injected with curdlan or PBS and culled after 1 wk to generate the data shown in I–L. **(I)** Frequency of indicated progenitors among live BM cells with curdlan or PBS (points show individual mice with mean, Mann-Whitney *U* test, representative of four independent experiments). **(J)** Absolute numbers of BM progenitors, mature erythroid cells (Ter119^+^), and neutrophils (NEUT) derived from one femur and one tibia in WT mice after injecting curdlan or PBS (points show individual mice with mean, Mann-Whitney *U* test, representative of four independent experiments). **(K)** Erythroid differentiation pathway for WT mice after injection of PBS or curdlan. Points represent mean with SD for *n* = five to six mice per group, and lines show LOWESS splines, Mann-Whitney *U* test, representative of four independent experiments. **(L)** Blood Hgb concentration in WT mice after injection of PBS or curdlan (points show individual mice with mean, Mann-Whitney *U* test, values pooled from two independent experiments). *, P < 0.05; **, P < 0.01; ***, P < 0.001; ****, P < 0.0001.

A clinical phenotype of chronic disease resembling SpA develops in SKG mice injected with the bacterial cell wall extract curdlan, a β-1,3-glucan that causes release of inflammatory cytokines from innate cells after binding to the receptor dectin-1 (Kim et al., 2016; Ruutu et al., 2012; Fig. 1 A). Over a period of 4 wk following injection of curdlan, such mice developed swelling of the paws and tarsal joints (ankles; Fig. 1, B and C), with associated weight loss (Fig. 1 D). The arthritis was associated with accumulation of mature (CD11b^+^Ly6G^+^) neutrophils in paws (Fig. 1 E), with the severity of infiltration correlating with clinical score, the extent of paw and tarsal swelling, and weight loss (Fig. 1 F and Fig. S1 A).

Patients with SpA have increased blood neutrophil counts (Mercan et al., 2016), and 15% have anemia at the time of diagnosis (Furst et al., 2013). We observed a similar reduction in blood hemoglobin (Hgb) concentration and RBC count in mice with SpA (Fig. 1 G). The Hgb concentration was not associated with changes in bodyweight (Fig. S1 B) but was unexpectedly positively correlated with change in paw size (Fig. 1 H). This latter finding could be related to development of dehydration in mice with more severe disease, spuriously increasing Hgb concentrations, or to concurrent inflammation of the small intestine (enteritis), a common feature of SpA in people (De Vos et al., 1996), which seemed to be associated with more severe anemia but milder paw swelling (Fig. 1 H).

These hematologic changes prompted us to ask whether there were associated perturbations in hematopoiesis in BM. Accordingly, when we plated BM cells in methylcellulose medium, we observed a lesser number of erythroid colonies (blast-forming units–erythroid [BFU-Es]) derived from cells of SpA mice after 10 d (Fig. 1 I) compared with healthy mice. Further characterization of BM HSPCs by flow cytometry using previously published definitions (Pietras et al., 2015; Pronk et al., 2007) revealed expansion of multipotent progenitor (MPP) and primitive hematopoietic stem cell (HSC) populations (Fig. S1 C), as we described previously (Regan-Komito et al., 2020). Downstream of these HSPCs, there was a significant decrease in the proportion of erythroid progenitors (CFU-erythroid [CFU-Es]) but a significant increase in myeloid progenitors (granulocyte macrophage progenitors [GMPs]) among the pool of lineage^neg^cKit^+^Sca1^neg^ progenitors (Fig. 1 J) and total live BM cells (Fig. 1 K). These changes were mirrored by a significant reduction in the absolute number of CFU-Es and a significant increase in GMPs (Fig. S1 D), because the total number of BM cells was unchanged (Fig. S1 E). Changes in progenitor populations were reflected in the mature cells of the BM, with a decreased proportion expressing the mature erythroid marker Ter119 in SpA, whereas the frequency of mature neutrophils was increased (Fig. 1, L and M; and Fig. S1 F).

To investigate changes in erythropoiesis further, we calculated the number of cells in the BM at successive stages of erythroid differentiation, beginning with committed progenitors (CFU-Es) and progressing to nucleated erythroblasts defined by characteristic surface markers (Fig. S1 G). When comparing these erythroid differentiation pathways for healthy mice and those with SpA, we confirmed that the rate and extent of erythroid differentiation were impaired in mice with chronic inflammation across early and late stages (Fig. 1 N).

With development of SpA, we also observed accumulation of erythroid progenitors in the spleen of inflamed mice (Fig. S1 H), but this was insufficient to prevent development of anemia (Fig. 1 G), indicating the functional output of this population did not compensate for loss of erythroid progenitors in the BM.

Taken together, these observations show that disease in SKG mice is associated with increased myelopoiesis and decreased erythropoiesis, resulting in similar hematologic changes to those described in people with SpA. To ensure these observations were not attributable to the mutation in *Zap70* that predisposes SKG mice to autoimmune responses, we confirmed that inflammation induced by intraperitoneal (IP) injection of curdlan in WT C57BL/6 mice produces the same hematopoietic phenotype after 1 wk (i.e., increased GMP and decreased CFU-E percentages and absolute numbers; Fig. S1, I and J) but does not induce clinical arthritis (data not shown). Whereas curdlan injection produced a similar inhibitory effect on the BM erythroid differentiation pathway (Fig. S1 K), the blood Hgb concentration was not decreased (Fig. S1 L), presumably because RBCs have a longer circulating half-life of ∼15–20 d in blood (Amireault et al., 2011; Van Putten, 1958) than the duration of these experiments in WT mice.

### The receptor for IL-33, ST2, is expressed preferentially and at high levels on erythroid progenitors

**Figure 2.**
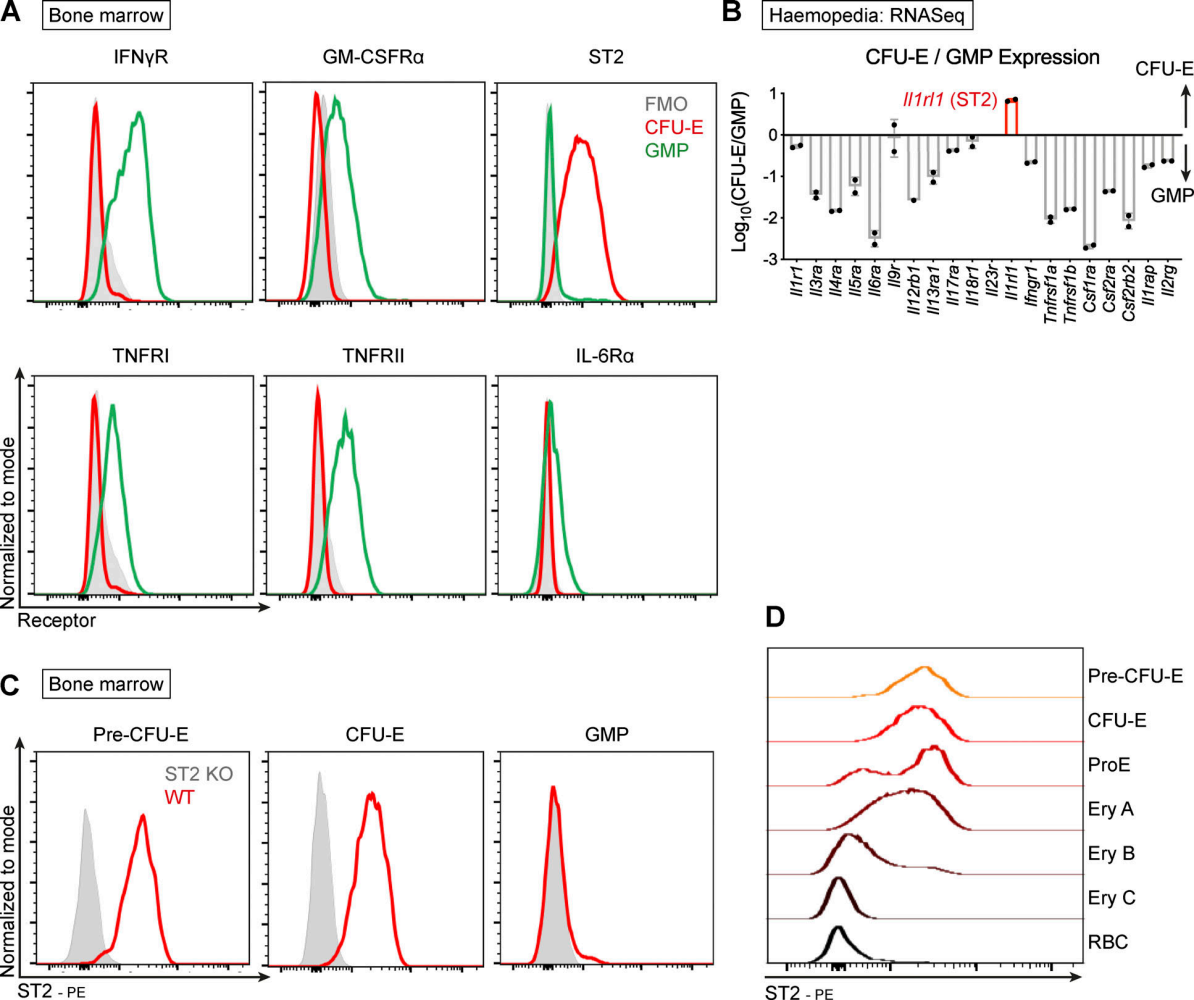
**The receptor for IL-33, ST2, is expressed preferentially on erythroid progenitors.** **(A)** Representative flow cytometric expression of cytokine receptors on CFU-E and GMP in BM of healthy SKG mice. FMO, fluorescence-minus-one. Representative of two independent experiments. **(B)** Ratio of transcript counts for indicated cytokine receptor genes in CFU-E and GMP by RNA sequencing (mean and SEM of two biological replicates). Data derived from Haemosphere database. **(C)** Representative flow cytometric expression of ST2 receptor on indicated progenitors in BM of ST2^−/−^ mice and WT mice bred in same facility, both on C57BL/6 background. Representative of two independent experiments. **(D)** Representative image of flow cytometric expression of ST2 on cells of the erythroid lineage of healthy SKG mice from least mature (pre–CFU-E) to most mature. Ery A, erythroblast A (Ter119^+^CD71^+^FSC^hi^); Ery B, erythroblast B (Ter119^+^CD71^+^FSC^lo^); Ery C, erythroblast C (Ter119^+^CD71^neg^FSC^lo^); ProE, proerythroblast (CD71^+^Ter119^int^); RBC, Ter119^+^CD71^neg^FSC^lo^. Representative of two independent experiments.

**Figure S2.**
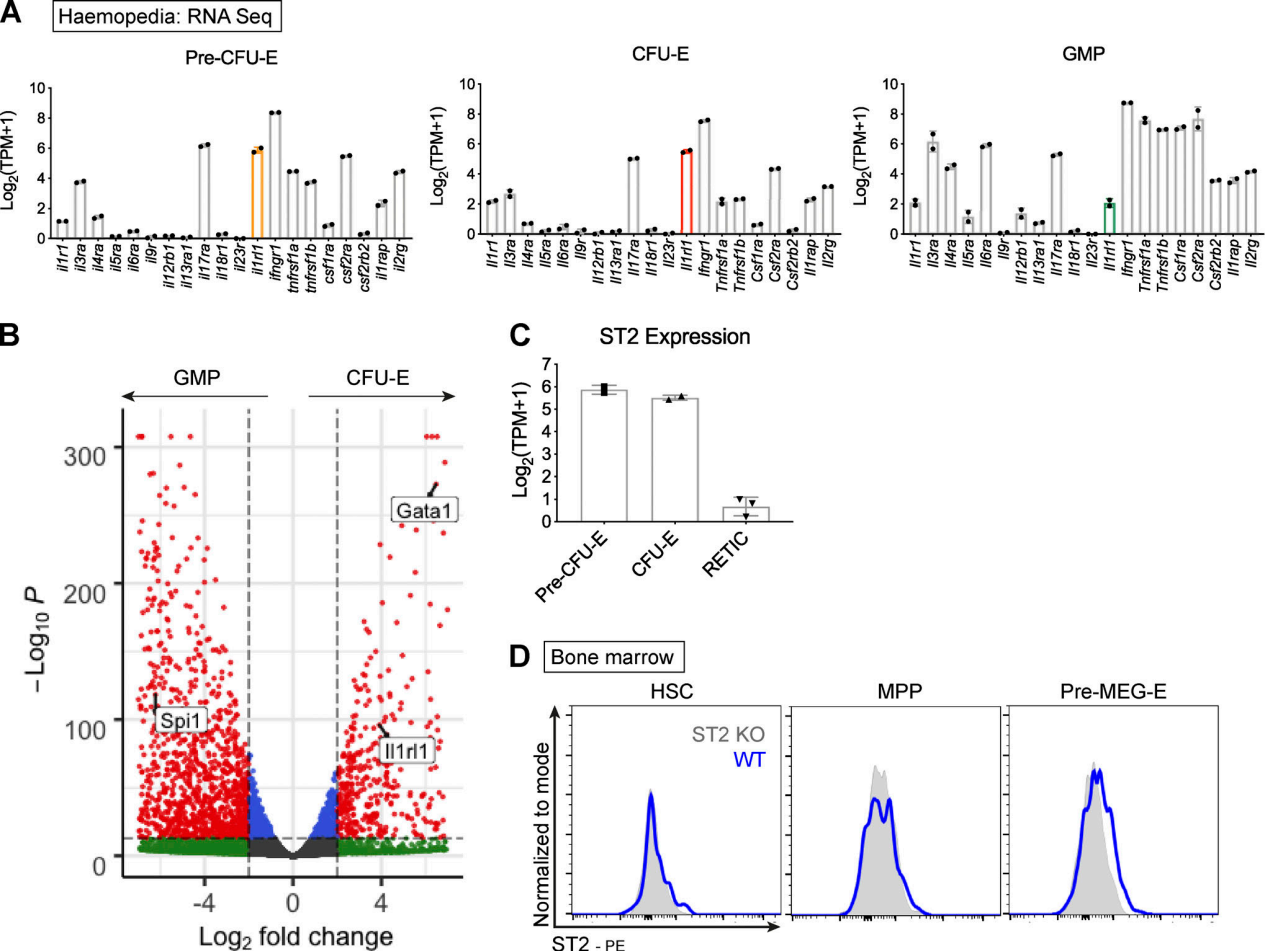
**Association between IL-33 and erythroid progenitors.** **(A)** Frequency of transcript counts for indicated cytokine receptor genes in indicated progenitor populations. Gene for the IL-33 receptor, ST2, is highlighted. Data derived from Haemosphere RNA-sequencing database of BM progenitors. Bars represent mean and SEM of two biological replicates. **(B)** Volcano plot comparing gene expression between GMP and CFU-E. The IL-33 receptor gene, *Il1rl1*, is highlighted, along with critical erythroid (*Gata1*) and myeloid (*Spi1*, PU.1) transcription factors. Data derived from comparison of two paired replicates from independent experiments in the Haemosphere database. **(C)** Frequency of transcript counts for *Il1rl1* (encoding ST2) among indicated progenitor populations. RETIC, reticulocyte. Bars represent mean and SEM of two biological replicates from the Haemosphere database. **(D)** Representative images of flow cytometric expression of the ST2 receptor on indicated progenitor populations in BM of WT and ST2^−/−^ (ST2 KO) mice. ST2^−/−^ mice were on C57BL/6 background; WT mice were bred under the same conditions. HSCs: Lineage^neg^, Sca1^+^, cKit^+^, CD150^+^, CD48^−^; MPPs: LSK, CD150^−^, CD48^+^; pre-MEG-E, premegakaryocyte-erythroid progenitor (Lineage^neg^, Sca1^−^, cKit^+^, CD41^−^, CD16/32^−^, CD105^−^, CD150^+^). Representative of two independent experiments.

AID is a syndrome of multifactorial etiology that accompanies several diseases, including SpA, rheumatoid arthritis, and inflammatory bowel disease (Nemeth and Ganz, 2014). Inflammatory cytokines may contribute to this phenomenon by altering lineage commitment of multipotent stem cells. However, we hypothesized that cytokines may have differential inhibitory effects on terminal differentiation of lineage-committed progenitors, limiting the output of some progenitors in the context of disease. To investigate this, we compared the surface expression of a panel of inflammatory cytokine receptors between committed myeloid GMP and erythroid CFU-E progenitors (Fig. 2 A). The receptor for the prototypical myeloid cytokine GM-CSF was preferentially expressed on GMP, but we were surprised to find the receptor for IL-33, ST2, at much higher levels on CFU-E (Fig. 2 A). Differential expression of receptor proteins was reflected in the transcriptome, with the *Il1rl1* gene (encoding ST2) being the only major proinflammatory cytokine receptor gene expressed at higher levels in CFU-Es compared with GMPs (Fig. 2 B and Fig. S2, A and B; Choi et al., 2019). We excluded the possibility of nonspecific staining in mice lacking the *Il1rl1* gene (ST2^−/−^; Fig. 2 C) and further showed that ST2 expression is highest on erythroid progenitors immediately after commitment to the lineage (i.e., pre–CFU-E and CFU-E) but decreases with progressive maturation (Fig. 2 D and Fig. S2 C), suggesting any effect of IL-33 in the erythroid lineage must be exerted while cells are differentiating. Importantly, the receptor was not expressed on primitive HSCs or MPPs upstream of the erythroid lineage (Fig. S2 D), reducing the likelihood of a lineage-instructive effect.

### IL-33 inhibits differentiation of erythroid progenitors

**Figure S3.**
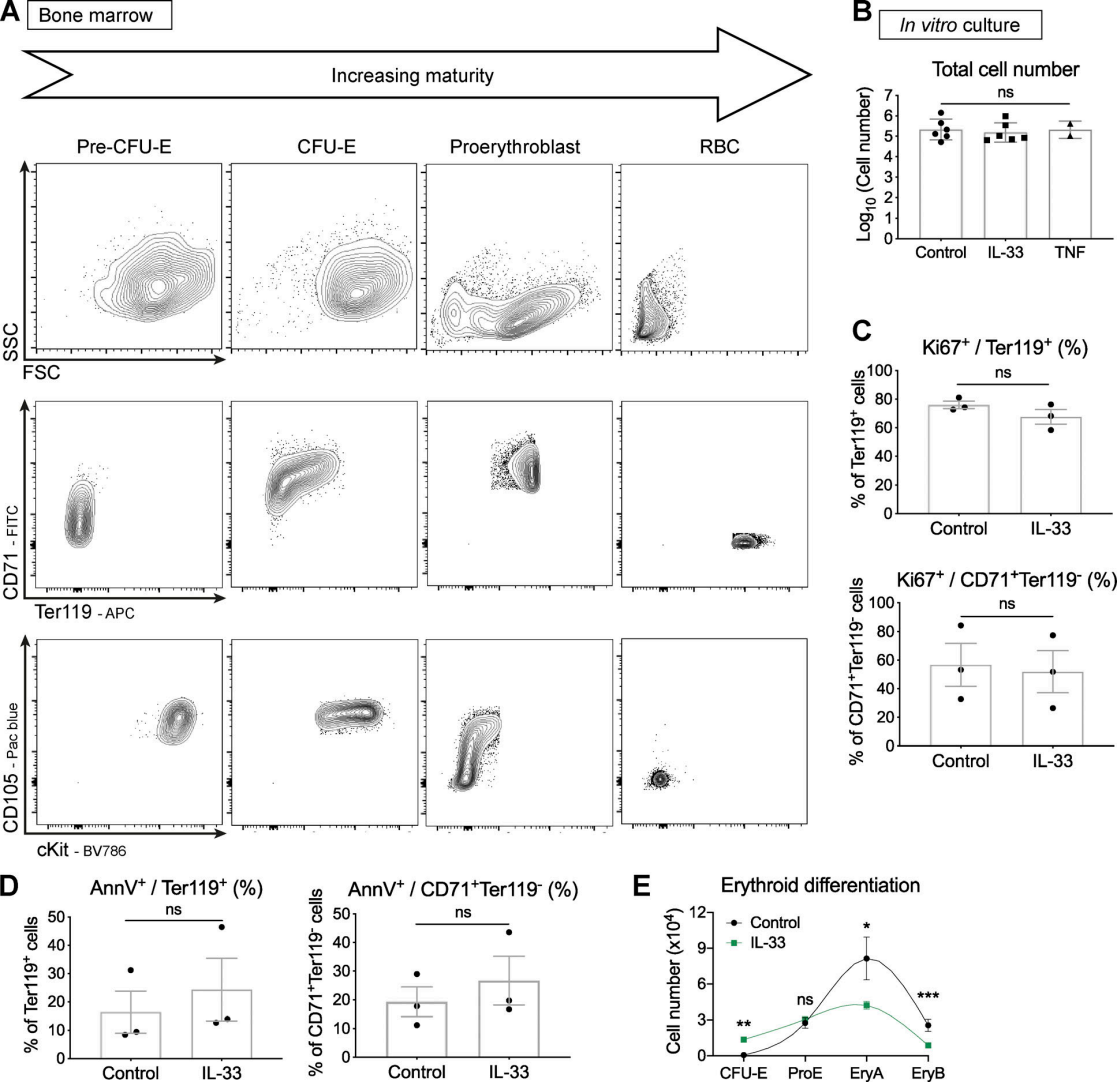
**IL-33 does not affect Ki67 or annexin V expression in murine erythroid progenitors in vitro.** **(A)** Representative flow cytometric images of indicated erythroid progenitors and RBCs from healthy SKG BM showing forward/side scatter characteristics (FSC and SSC) and expression of maturation markers in erythroid cells at progressive stages of maturation. Representative of four independent experiments. **(B)** Absolute number of live cells derived from cultures of 2,000 sorted pre–CFU-E after 4 d. Points represent mean of technical triplicates from *n* = two (TNF) or six (IL-33) independent experiments; bars represent mean with SEM. Groups compared by one-way ANOVA with Tukey’s test. **(C)** Frequency of indicated cultured cell populations expressing Ki67. Points represent mean of technical triplicates from *n* = three independent experiments. Bars represent mean with SEM, unpaired *t* test. **(D)** Frequency of indicated cultured cell populations expression annexin V. Points represent mean of technical triplicates from *n* = three independent experiments. Bars represent mean with SEM, unpaired *t* test. **(E)** Erythroid differentiation pathway for cultured pre–CFU-E. Points represent mean and SD of technical triplicates. Lines show locally weighted scatterplot smoothing (LOWESS) splines, representative of four independent experiments. *, P < 0.05; **, P < 0.01; ***, P < 0.001.

**Figure 3.**
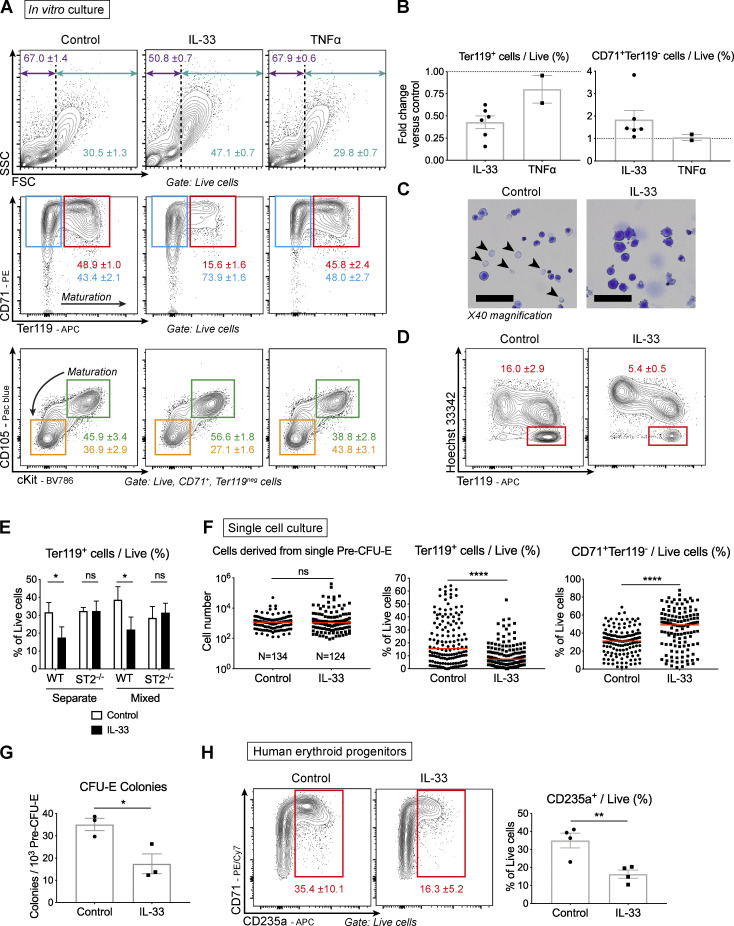
**IL-33 inhibits differentiation of murine and human erythroid progenitors in vitro.** **(A)** Representative images of cells from culture of FACS-sorted pre–CFU-E cells with IL-33 or TNF-α where indicated. Figures show mean and SD of technical triplicates. Top: Frequency of small (purple) and large cells (blue). Middle: Frequency of cells expressing Ter119 and CD71 (red) and only CD71 (blue). Bottom: Frequency of CD71^+^Ter119^−^ cells expressing CD105 and cKit (green) or negative for both (orange). Representative of two (TNF-α) or six (IL-33) independent experiments. FSC, forward scatter characteristics; SSC, side scatter characteristics. **(B)** Fold change in frequency of Ter119^+^ cells (left) or CD71^+^Ter119^neg^ cells (right) in culture with IL-33 or TNF-α compared with control (points show *n* = two [TNF-α] or six [IL-33] independent experiments, *n* = three replicates per group per experiment, with mean and SEM). **(C)** Representative photomicrographs of cells cultured for 4 d, stained with Kwik Diff. Arrowheads indicate enucleated RBCs. Scale bars = 200 µm. Representative of two independent experiments. **(D)** Representative flow cytometric images showing proportion of enucleated cells (Ter119^+^, Hoechst^neg^) among live cells. Figures show mean and SD of technical triplicates, representative of two independent experiments. **(E)** Frequency of Ter119^+^ cells when plating cells from Ub-GFP and ST2^−/−^ mice alone or in a 1:1 mixture, with or without IL-33 (mean and SD of technical triplicates, two-way ANOVA with Sidak’s multiple comparison test, representative of two independent experiments). **(F)** Total cell number (left) and frequencies of indicated cells (right) from single pre–CFU-Es FACS sorted into 96-well plates and harvested after 10 d (points show values for single cells with median, Mann-Whitney *U* test, pooled from four independent experiments). **(G)** Frequency of CFU-E colonies obtained 2 d after plating 1,000 FACS-sorted pre–CFU-E cells in methylcellulose medium, ±IL-33 (mean of *n* = three independent experiments, *n* = four to five mice per group per experiment, with mean and SEM, unpaired *t* test). **(H)** Representative images and frequency of CD235a^+^ cells from FACS-sorted CD34^+^CD71^+^ progenitors from human blood cones cultured for 8–9 d ±IL-33 (figures show mean and SD of technical triplicates. Points in graph show mean of *n* = four independent cones, *n* = three replicates per cone, with mean and SEM, Mann-Whitney *U* test). *, P < 0.05; **, P < 0.01; ****, P < 0.0001.

With the receptor ST2 expressed on erythroid progenitors, we hypothesized that IL-33 would exert a direct effect on these cells that might promote development of AID. To investigate this, we optimized a culture system by sorting progenitors at the earliest stage after commitment to the erythroid lineage (pre–CFU-E) by FACS from adult BM. We cultured these cells in proerythroid medium supplemented with stem cell factor and EPO, with or without IL-33 or TNF-α, for 4 d before assessing cells according to their size and expression of Ter119. With progressive differentiation, these pre–CFU-Es become smaller, up-regulate and then down-regulate the transferrin receptor CD71, lose cKit and CD105, and express Ter119 (Fig. S3 A; Chen et al., 2009; Pop et al., 2010; Tusi et al., 2018). In control conditions and with TNF-α, the majority of cells were small and had little granularity, but, in the presence of IL-33, a greater proportion of cells kept a larger, blast-like profile (Fig. 3 A), as observed in primary BM pre–CFU-Es (Fig. S3 A). Similarly, IL-33 caused a striking reduction in the proportion of cells expressing Ter119 and, even among the less mature CD71^+^Ter119^−^ cells, IL-33 also prevented down-regulation of the surface receptor cKit, which is normally lost with maturation (Fig. 3, A and B; and Fig. S3 A). In the terminal stages of maturation, the nucleus is extruded from progenitors to form reticulocytes. In our culture system, addition of IL-33 decreased the number of these enucleated cells observed cytologically (Fig. 3 C) and by staining for nuclear material in Ter119^+^ cells by flow cytometry (Fig. 3 D).

HSPCs can secrete cytokines themselves in response to pathogen derivatives and cytokines (Zhao et al., 2014), possibly superseding the effects of the original stimulus by autocrine signaling. To evaluate whether the observed effects of IL-33 in vitro were dependent on other factors, we cultured pre–CFU-Es from mice expressing GFP under the control of the ubiquitin c promoter (Ub-GFP), which is still expressed in maturing RBCs, and from ST2^−/−^ mice, alone or in a 1:1 mixture. As expected, IL-33 had no effect on the proportion of Ter119^+^ cells derived from ST2^−/−^ cells when cultured alone (Fig. 3 E). Importantly however, the frequency of Ter119^+^ cells was also unchanged in ST2^−/−^ cells in mixed cultures with Ub-GFP cells that were affected by IL-33, indicating this was a direct effect mediated through the ST2 receptor (Fig. 3 E).

Addition of IL-33 did not alter total cell yield from culture (Fig. S3 B) or the proportion of cells expressing markers of proliferation (Ki67; Fig. S3 C) or apoptosis (annexin V; Fig. S3 D), leading us to suspect it was primarily suppressing the differentiation of erythroid progenitors, as also suggested by the difference in erythroid differentiation pathways (Fig. S3 E). To test this, we separated the effects of IL-33 on proliferation and differentiation by sorting single pre–CFU-E cells and culturing them for 10 d before assessing the output by flow cytometry. We found the total cell yield was similar, but the proportion of Ter119^+^ cells was decreased by addition of IL-33, as it was in bulk cultures, confirming a primary effect on erythroid differentiation (Fig. 3 F). Functionally, this effect was recapitulated by decreased formation of CFU-E colonies when FACS-sorted pre–CFU-Es were plated in methylcellulose medium with IL-33 (Fig. 3 G).

Finally, we asked whether this effect of IL-33 on erythroid progenitors was common to other species by isolating CD34^+^CD71^+^ progenitors (Mori et al., 2015) from human blood leukoreduction cones. When culturing these cells for 8–9 d in similar conditions, inclusion of IL-33 decreased the frequency of mature erythroid cells expressing glycophorin A (CD235a, similar to murine Ter119; Kina et al., 2000) by approximately twofold (Fig. 3 H).

Collectively, these observations show that IL-33 suppresses differentiation of erythroid progenitors in a direct and cell-intrinsic manner, in a mechanism that appears to be common to mice and humans.

### IL-33 is increased in the BM niche with inflammation

**Figure 4.**
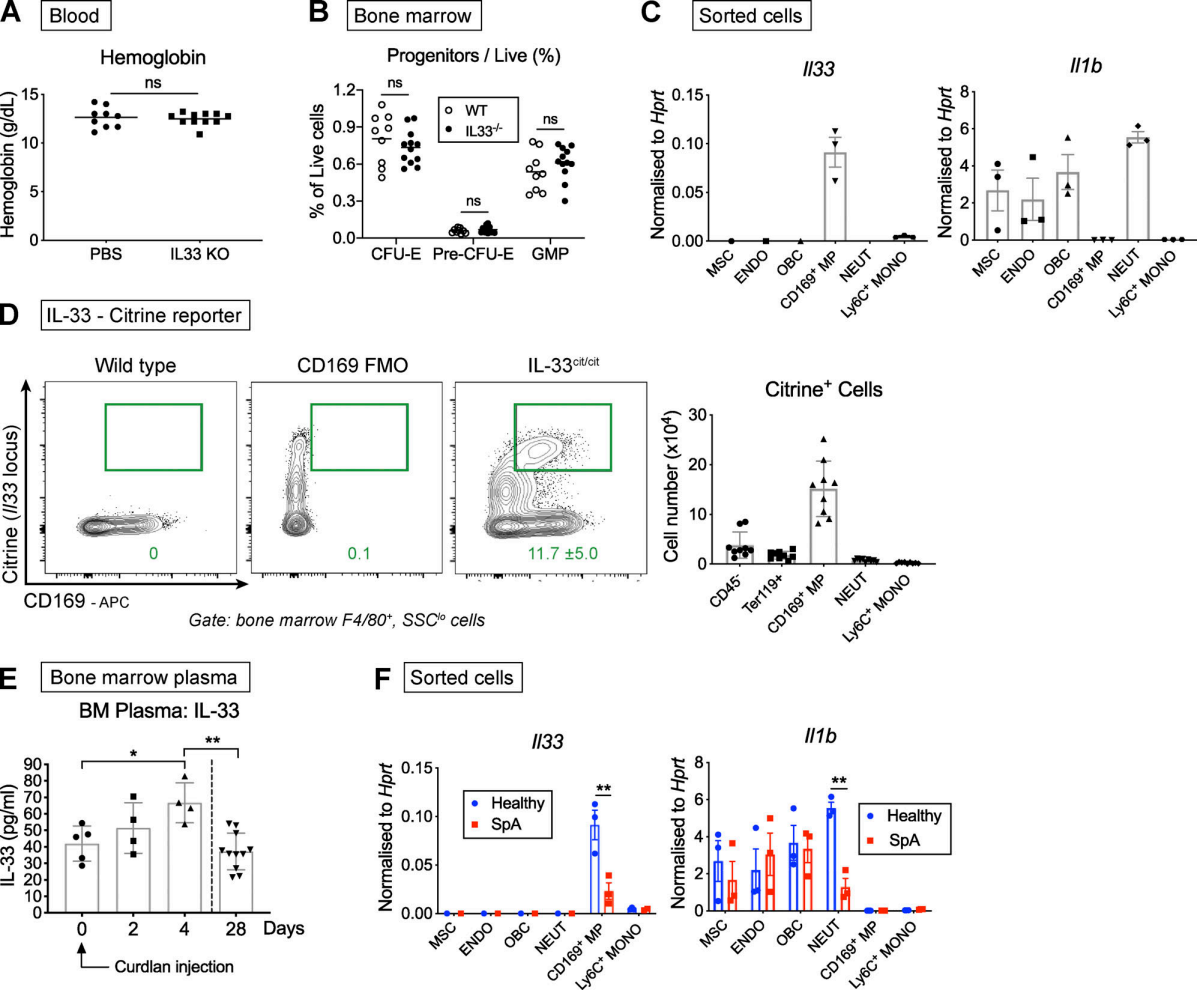
**IL-33 is produced in the BM niche and increased with inflammation.** **(A)** Blood Hgb concentration measured in IL-33^−/−^ and WT mice (points show individual mice with mean, Mann-Whitney *U* test, representative of two independent experiments). IL-33^−/−^ mice backcrossed for eight generations onto same C57BL/6 colony from which WT mice derived. **(B)** Proportion of indicated BM progenitors among live BM cells (points show individual mice with mean, two-way ANOVA with Sidak’s multiple comparison test, representative of four independent experiments). **(C)** Expression of indicated genes by RT-qPCR in cells sorted by FACS from flushed (CD169^+^ macrophage [MP], neutrophil [NEUT], Ly6C^+^ monocyte [MONO]) or crushed and digested (mesenchymal stromal/stem cells [MSC], endothelium [ENDO], and osteoblastic lineage cell [OBC]) bones of healthy SKG mice. Expression normalized to *Hprt* (mean and SEM of *n* = three independent experiments, *n* = two mice per group per experiment, unpaired *t* test). **(D)** Left: Representative flow cytometric images of citrine (GFP) expression in CD169^+^F4/80^+^ BM macrophages in WT mice ±CD169 staining and in mice expressing citrine at the *Il33* locus (IL-33^cit/cit^). IL-33^cit/cit^ mice backcrossed for eight generations to the same C57BL/6 colony from which WT mice derived (mean and SD, *n* = eight, representative of two independent experiments). Right: Absolute number of citrine^+^ cells derived from one femur and one tibia of IL-33^cit/cit^ mice (points show individual mice with mean and SD, pooled from two independent experiments). **(E)** Concentration of IL-33 in BM plasma of littermate SKG mice before (day 0) and indicated time after curdlan injection (points show individual mice with mean and SD, one-way ANOVA with Tukey’s test, representative of two independent experiments). **(F)** Expression of indicated genes by RT-qPCR in indicated populations sorted by FACS as for C in healthy SKG mice and littermates with SpA. Expression normalized to *Hprt* (mean and SEM of *n* = three independent experiments, *n* = two mice per group per experiment, two-way ANOVA with Sidak’s multiple comparison test). *, P < 0.05; **, P < 0.01.

Having detected expression of its receptor at high levels on erythroid progenitors, we asked whether IL-33 had a role in erythropoiesis in healthy mice. However, mice lacking the *Il33* gene had normal Hgb concentrations compared with WT counterparts (Fig. 4 A) and similar proportions of lineage-committed progenitors (Fig. 4 B). To establish whether IL-33 is produced in the BM niche, we sorted major populations of hematopoietic and stromal cells by FACS using published definitions (Chow et al., 2013; Pinho et al., 2013; Schepers et al., 2013) and found the *Il33* gene, unlike the gene encoding the related cytokine IL-1β, was preferentially expressed in CD169^+^F4/80^+^ macrophages, previously described as erythroblastic island macrophages that maintain close physical contact with erythroid progenitors in the BM and contribute to their retention and differentiation (Fig. 4 C; Chow et al., 2013). Using mice expressing a citrine reporter in place of the *Il33* gene, we found these CD169^+^ macrophages were the most abundant IL-33 producers in the BM (Fig. 4 D), exceeding the numbers of IL-33^+^ Ter119^+^ and CD45^−^ stromal cells described previously (Kenswil et al., 2018; Wei et al., 2015).

Collectively, these observations show that IL-33 is produced in healthy mice within the BM niche inhabited by erythroid progenitors but is dispensable for maintenance of a normal erythroid compartment. However, after injecting curdlan to trigger SpA, the concentration of IL-33 measured in BM plasma increased rapidly (Fig. 4 E), whereas its expression in CD169^+^ macrophages decreased in mice with SpA (Fig. 4 F). This suggests increased IL-33 production in inflamed tissues, which we described previously in the SKG model (Regan-Komito et al., 2020), may supersede local production in the BM niche, leading us to hypothesize that IL-33 could be a factor regulating erythropoiesis after curdlan injection.

### IL-33 is required for curdlan-induced suppression of erythropoiesis

**Figure 5.**
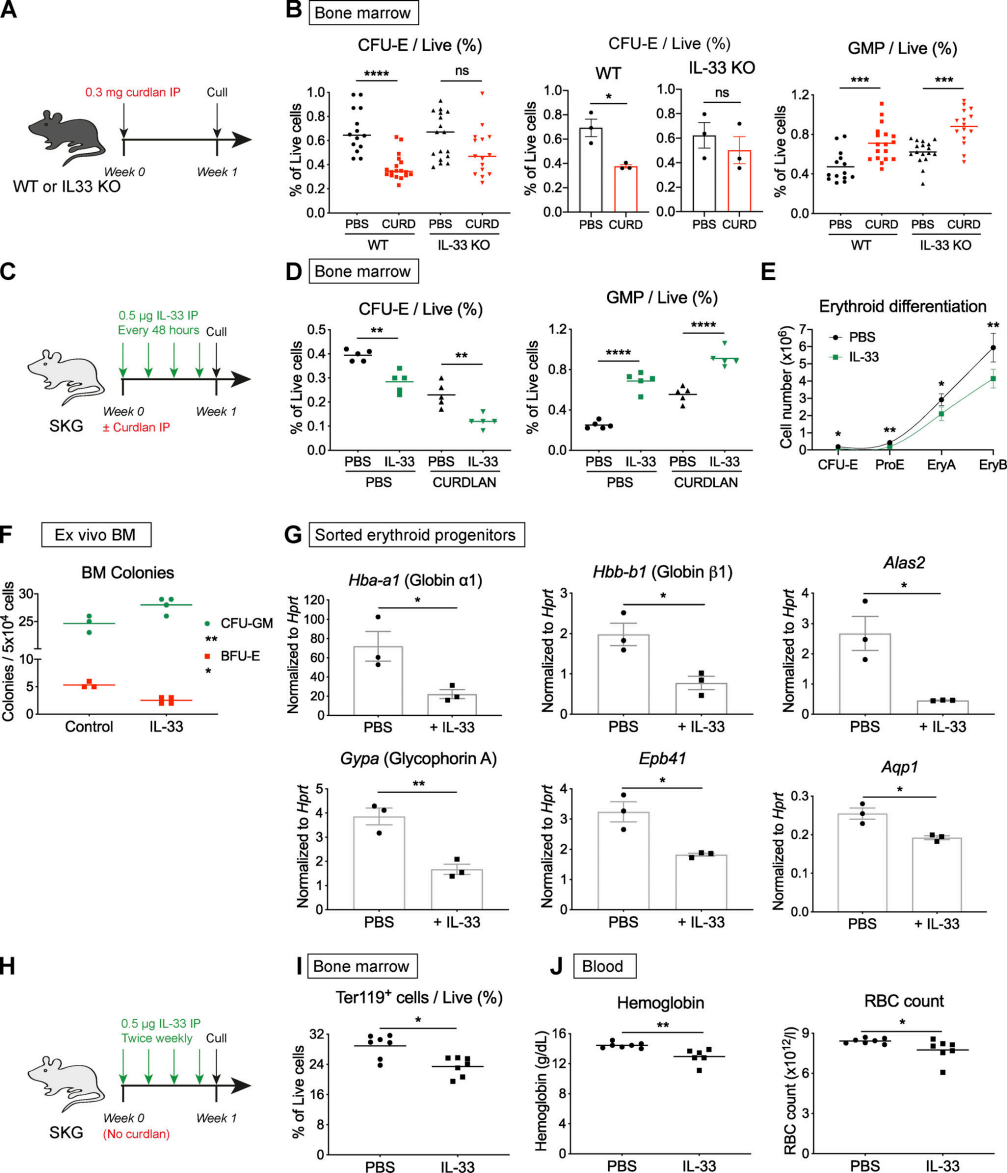
**IL-33 is implicated in suppression of erythropoiesis during chronic inflammation.** **(A)** Schematic diagram showing injection of curdlan (CURD) or PBS in IL-33^−/−^ mice backcrossed for eight generations onto same C57BL/6 colony from which WT control mice derived, before culling after 1 wk to obtain data shown in B. **(B)** Frequency of CFU-E (left, middle) and GMP (right) among live BM cells. For CFU-Es, results are presented as pooled values from three independent experiments (left; points show individual mice with mean, one-way ANOVA with Tukey’s test) or as a summary of the same three independent experiments (middle) with mean and SEM (*n* = four to six mice per group per experiment, unpaired *t* test). For GMP, points show individual mice with mean pooled from three independent experiments. Groups compared by one-way ANOVA with Tukey’s test. **(C)** Schematic diagram showing injection of SKG mice with recombinant murine IL-33, after injection of PBS or curdlan in littermate controls, before culling after 1 wk to obtain data shown in D, E, and G. **(D)** Frequency of indicated progenitors among live BM cells (points show individual mice with mean, one-way ANOVA with Tukey’s test, representative of three independent experiments). **(E)** Erythroid differentiation pathway (mean and SD for *n* = five to six mice per group with locally weighted scatterplot smoothing (LOWESS) spline, Mann-Whitney *U* test, representative of three independent experiments). **(F)** Frequency of indicated colonies from whole BM from healthy SKG mice in methylcellulose medium ±IL-33 after 10 d (mean and SD from *n* = three to four mice per group, two-way ANOVA with Sidak’s multiple comparison test, representative of two independent experiments). **(G)** Gene expression by RT-qPCR in FACS-sorted pre–CFU-Es from BM of healthy SKG mice and littermate controls injected with IL-33 (mean and SEM of *n* = three independent experiments, *n* = two mice per group per experiment, unpaired *t* test). **(H)** Schematic diagram showing injection of SKG mice with IL-33 or PBS in littermate controls over 4 wk to obtain data shown in I and J. **(I)** Frequency of erythroid (Ter119^+^) cells among live BM cells (points show individual mice with mean, Mann-Whitney *U* test, representative of two independent experiments). **(J)** Blood Hgb concentration (left) and RBC count (right). Points show individual mice with mean, Mann-Whitney *U* test, representative of two independent experiments. *, P < 0.05; **, P < 0.01; ***, P < 0.001; ****, P < 0.0001.

**Figure S4.**
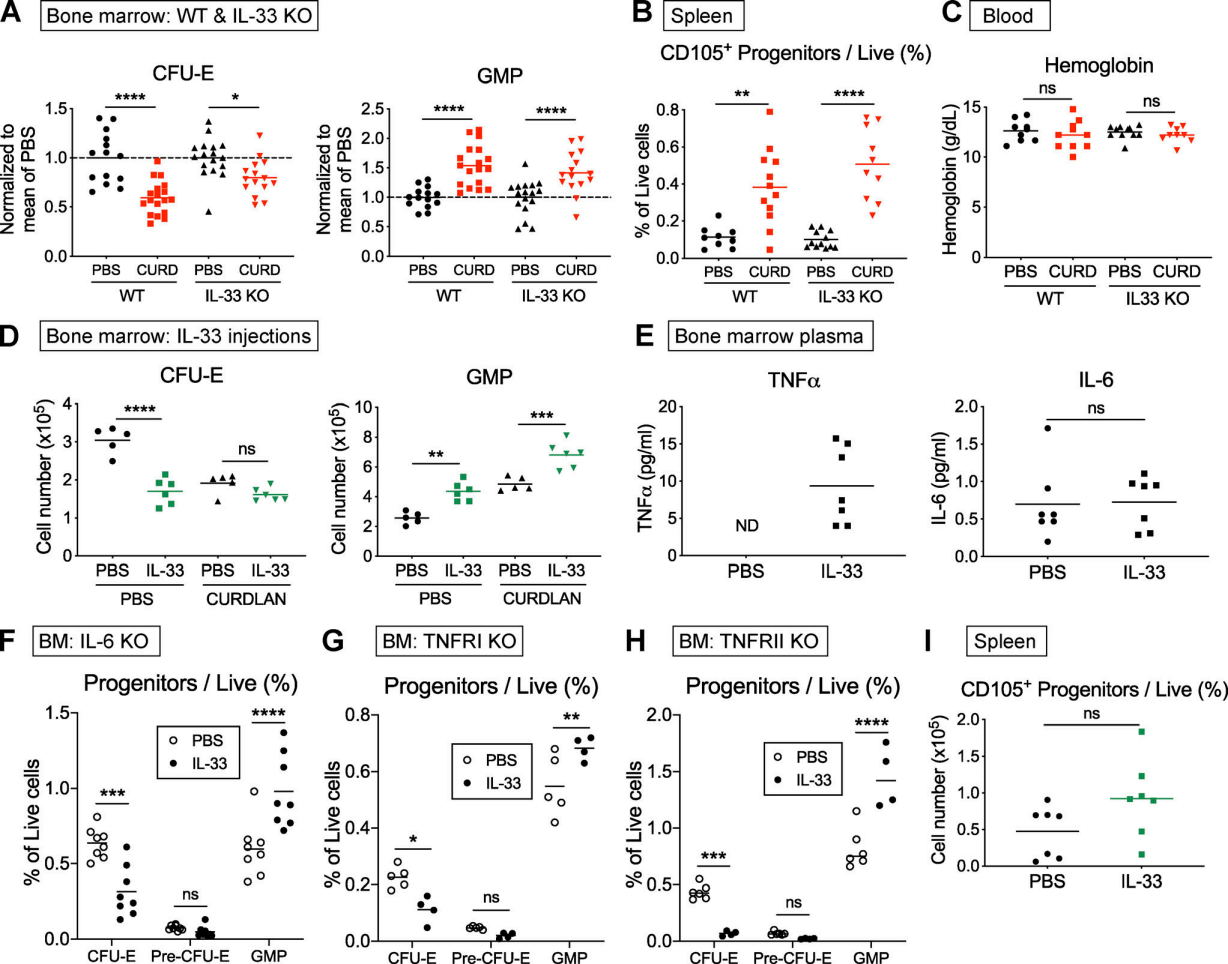
**Role of IL-33 in suppression of erythropoiesis in vivo.** **(A)** Absolute number of indicated progenitor populations in BM of WT and IL-33^−/−^ mice injected with PBS or curdlan and culled after 1 wk. IL-33^−/−^ were backcrossed for eight generations onto the same colony of WT mice used as controls. Data pooled from three independent experiments, with values normalized to mean PBS values for each experiment. Points show individual mice with mean, one-way ANOVA with Tukey’s test. **(B)** Frequency of erythroid progenitors among live splenocytes in indicated strains with indicated treatment (points show individual mice with mean, one-way ANOVA with Tukey’s test, values pooled from two independent experiments). **(C)** Blood Hgb concentration in indicated strains with indicated treatment (points represent individual mice with mean, one-way ANOVA, values pooled from two independent experiments). **(D)** Absolute number of indicated progenitor populations in BM of SKG littermate mice injected with IL-33 for 1 wk after injection of PBS or curdlan (points represent individual mice with mean, one-way ANOVA with Tukey’s test, representative of three independent experiments). **(E)** Concentration of indicated cytokines measured in BM plasma of SKG littermate mice injected with PBS or IL-33 for 1 wk. Points represent individual mice, and bars indicate mean. Groups were compared with Mann-Whitney *U* test. ND, not detected. Representative of two independent experiments. **(F)** Proportion of indicated BM progenitors among live cells in littermate IL-6^−/−^ mice injected with recombinant murine IL-33 or PBS for 1 wk before culling (points show individual mice with mean, two-way ANOVA with Sidak’s multiple comparison test, values pooled from two independent experiments). **(G)** Proportion of indicated BM progenitors among live cells in littermate TNFRI^−/−^ mice injected with PBS or recombinant murine IL-33 for 1 wk before culling (points show individual mice with mean, two-way ANOVA with Sidak’s multiple comparison test, representative of two independent experiments). **(H)** Proportion of indicated BM progenitors among live cells in littermate TNFRII^−/−^ mice injected with PBS or recombinant murine IL-33 for 1 wk before culling (points show individual mice with mean, two-way ANOVA with Sidak’s multiple comparison test, representative of two independent experiments). **(I)** Frequency of erythroid progenitors among live splenocytes in littermate SKG mice injected with PBS or IL-33 for 4 wk (points represent individual mice with mean, Mann-Whitney *U* test, representative of two independent experiments). *, P < 0.05; **, P < 0.01; ***, P < 0.001; **** P < 0.0001.

IL-33 suppressed differentiation of erythroid progenitors in vitro and was increased in BM after induction of inflammation. Against this backdrop, we investigated the functional role of IL-33 in AID by injecting curdlan in mice lacking the *Il33* gene (IL-33^−/−^) and matched WT C57BL/6 controls (Fig. 5 A). Whereas the frequency of erythroid progenitors decreased significantly in WT mice with this treatment, there was no significant reduction in CFU-Es in IL-33^−/−^ mice receiving either PBS or curdlan when results from individual mice were pooled or compared across independent experiments (Fig. 5 B), with a less striking difference in the number of cells (Fig. S4 A). Conversely, the frequency of myeloid GMPs increased in both strains with induction of inflammation (Fig. 5 B and Fig. S4 A), indicating the effect of IL-33 was specific to the erythroid lineage. As in SKG mice, curdlan caused expansion of the erythroid progenitor pool in the spleens of mice of both strains (Fig. S4 B), but we could not determine the functional impact of this extramedullary hematopoiesis because the blood Hgb concentration was unaltered in either strain (Fig. S4 C), presumably because the length of the experiment was again shorter than the RBC half-life.

Having shown it was necessary for curdlan-induced suppression of BM erythropoiesis, we next asked whether administration of IL-33 alone would be sufficient to produce the same hematopoietic changes as curdlan. We therefore injected SKG mice with IL-33 over 1 wk (Fig. 5 C); this treatment caused marked reductions in the frequency and absolute number of erythroid progenitors to a similar extent as observed with curdlan injection (Fig. 5 D and Fig. S4 D). Notably, divergence between erythroid differentiation pathways of healthy and IL-33–injected mice caused a more marked deficiency in more mature Ter119^+^ cells (Fig. 5 E), as was apparent in cultures of primary cells exposed to IL-33 (Fig. 3 A and Fig. S3 E). Changes in BM cell numbers were also reflected in functional differences in colony formation, with an increased number of myeloid (CFU-granulocyte macrophage) and decreased number of erythroid (BFU-E) colonies observed with addition of IL-33 to methylcellulose medium (Fig. 5 F).

In mast cells and eosinophils, IL-33 causes production of other proinflammatory cytokines that may suppress erythropoiesis, including TNF-α and IL-6 (Lunderius-Andersson et al., 2012). Indeed, we found the concentration of TNF-α, but not IL-6, was increased in BM plasma of mice injected with IL-33 (Fig. S4 E). To determine whether these cytokines were required, we injected IL-33 in mice lacking IL-6 or the receptors for TNF-α (TNFRI and TNFRII), but, in all three strains, IL-33 produced similar suppression of erythropoiesis (Fig. S4, F–H), indicating they were not required to produce those effects.

At a cellular level, there was a striking reduction in expression of the genes encoding the adult globin chains (*Hbb-b1* and *Hbb-a1*), forming the proteinaceous component of Hgb, in FACS-sorted pre–CFU-Es from mice exposed to IL-33 for 1 wk compared with controls (Fig. 5 G). There were similar reductions in expression of other genes required for production of mature RBCs, including those encoding the heme enzyme aminolevulinic acid synthase (*Alas2*) and the major surface proteins glycophorin A (*Gypa*), erythroid band protein 4.1 (*Epb41*), and aquaporin 1 (*Aqp1*; Fig. 5 G). With prolonged exposure to IL-33 over 4 wk (Fig. 5 H), these changes in cellular activity resulted in a decreased frequency of Ter119^+^ erythroid cells in BM (Fig. 5 I) and decreased Hgb concentrations and RBC counts in peripheral blood (Fig. 5 J). These changes were dependent on inhibition of BM erythropoiesis, because there was no significant change in the frequency of erythroid progenitors in the spleen (Fig. S4 I).

Taken together, these observations demonstrate that IL-33 is both necessary and sufficient for the full extent of curdlan-induced suppression of erythropoiesis. Remarkably, injection of IL-33 alone decreased expression of globin and heme synthesis enzyme genes, resulting in decreased blood Hgb with prolonged exposure.

### The effects of IL-33 in erythroid progenitors are dependent on NF-κB

**Figure 6.**
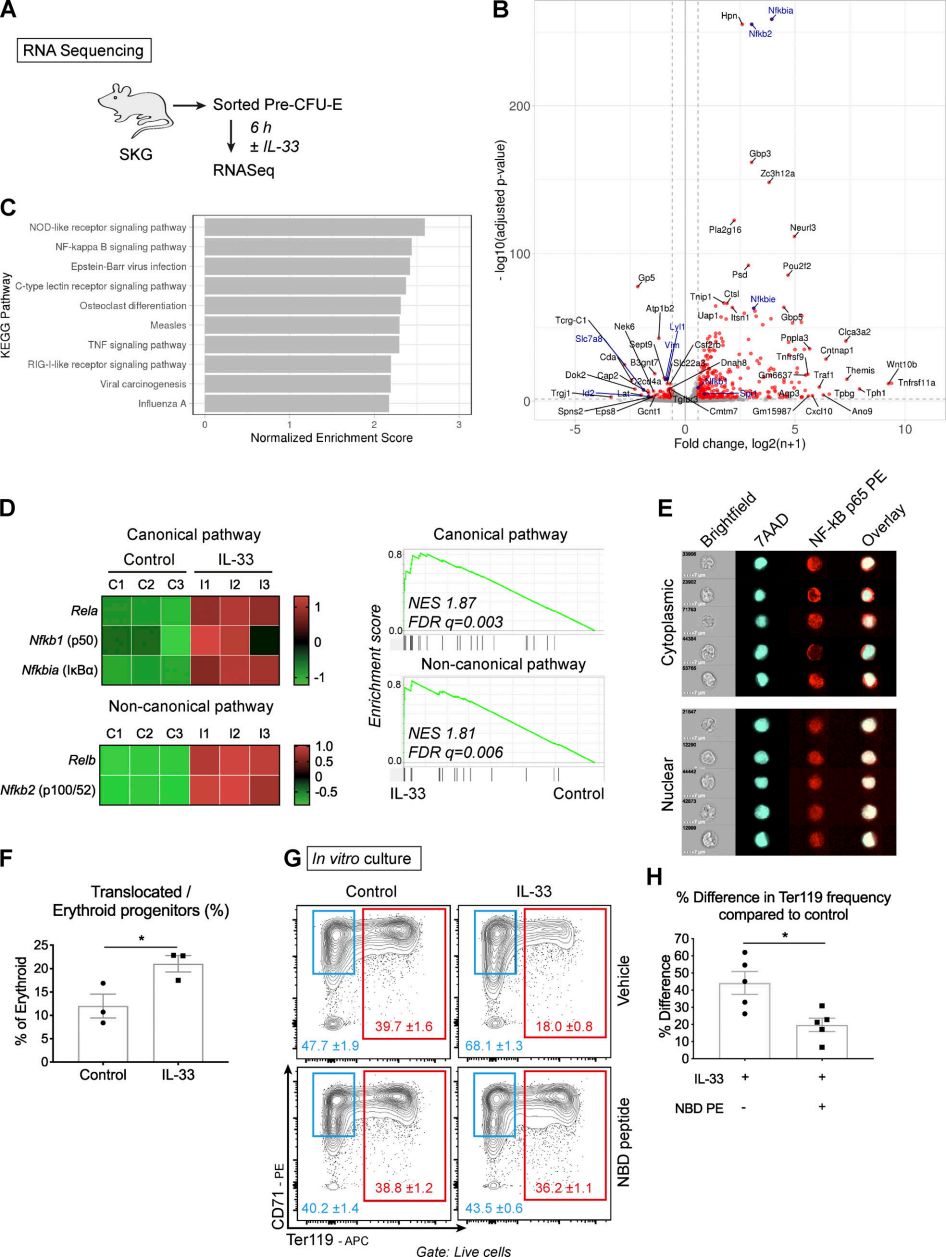
**IL-33–mediated suppression of erythropoiesis is dependent on NF-κB signaling.** **(A)** In three independent experiments, pre–CFU-Es were sorted from six SKG mice by FACS and cultured ±IL-33 for 6 h. RNA was extracted for sequencing. **(B)** Volcano plot showing differential expression of genes between conditions (red points show 1.5-fold up-regulation, adjusted P ≤ 0.01); selected members of the NF-κB pathway and functional erythroid genes are highlighted in blue. **(C)** Bar graph showing the 10 most enriched KEGG pathways among up-regulated genes in pre–CFU-Es with exposure to IL-33. **(D)** Left: Heatmaps showing differential expression of selected genes in canonical and noncanonical NF-κB pathways in pre–CFU-E, expressed as row-normalized z scores. Columns show paired replicates from *n* = three independent experiments, each *n* = six mice. Right: Enrichment plots generated by GSEA using gene sets for NF-κB canonical and noncanonical pathways. FDR q, false discovery rate q value; NES, normalized enrichment score. **(E)** Representative images of single erythroid progenitors (Lineage^neg^, cKit^+^, CD105^+^) by ImageStream cytometry, showing nuclear staining with 7AAD and NF-κB p65 in the cytoplasm or nucleus. Representative of three independent experiments. **(F)** Proportion of erythroid progenitors with nuclear p65 after exposure to IL-33 for 30 min where indicated (mean of *n* = three independent experiments, *n* = two replicates per group per experiment, with mean and SEM, unpaired *t* test). **(G)** Representative flow cytometric images of pre–CFU-Es cultured ±IL-33 and NBD peptide (figures show mean and SD of technical triplicates, representative of five independent experiments). **(H)** Difference in percentage of Ter119^+^ cells compared with control in pre–CFU-Es cultured as in G (mean of *n* = five independent experiments, *n* = three replicates per group per experiment, with mean and SEM, unpaired *t* test). *, P < 0.05.

**Figure S5.**
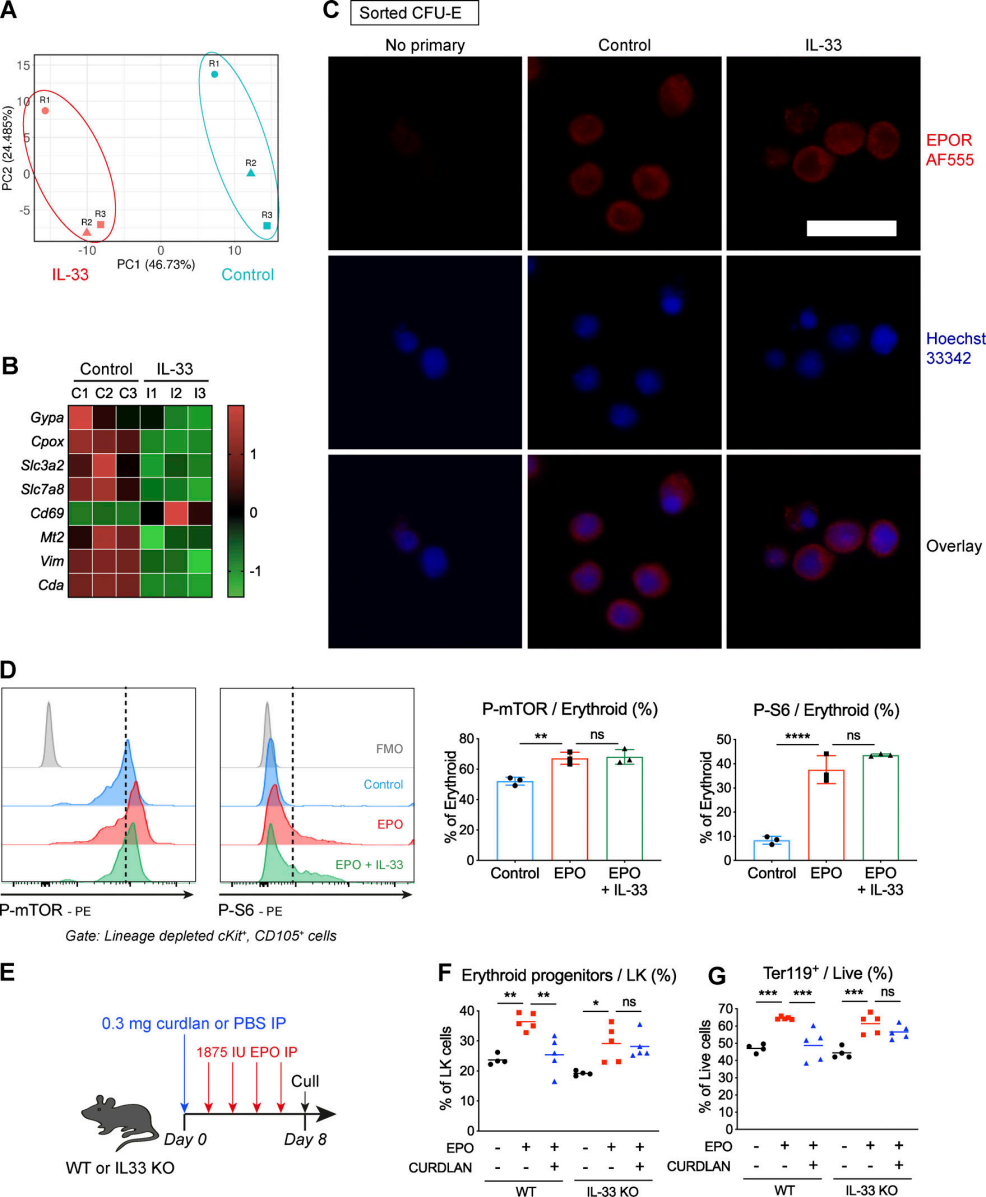
**IL-33 decreases expression of genes required for terminal erythroid maturation.** **(A)** Plot of principal components (PCs) showing clustering of control and IL-33–treated samples from *n* = three independent experiments (labeled R1–R3) based on differential gene expression from RNA sequencing analysis described in Fig. 6 A. **(B)** Heatmap showing differential expression of selected genes required for terminal erythroid differentiation in pre–CFU-Es by RNA sequencing, expressed as row-normalized z scores. Columns represent paired biological replicates from *n* = three independent experiments. **(C)** Representative fluorescence images of FACS sorted CFU-Es incubated with IL-33 or medium for 12 h and then stained for EPO-R and Hoechst nuclear stain. Bottom row shows overlay of single-color images. Scale bar = 25 µm. Representative of two independent experiments. **(D)** Representative images showing flow cytometric expression of indicated phosphorylated proteins in erythroid progenitors exposed to medium or IL-33 and then stimulated with EPO. Graphs show proportion of erythroid progenitors expressing indicated phosphorylated proteins. Points represent technical triplicates, and bars represent mean and SD; one-way ANOVA with Tukey’s test, representative of two independent experiments. mTOR, mammalian target of rapamycin. **(E)** Schematic diagram showing WT or IL33^−/−^ mice injected with curdlan or PBS, then injected four times with EPO IP as indicated before culling to produce data shown in F and G. IL-33^−/−^ mice were backcrossed for eight generations onto the same colony of WT mice from which controls were used. **(F)** Frequency of CD105^+^ erythroid progenitors (i.e., CFU-Es and pre–CFU-Es) among lineage^neg^cKit^+^Sca1^neg^ cells in BM. Points represent individual mice, lines represent mean. Groups were compared by one-way ANOVA with Tukey’s test, representative of two independent experiments. **(G)** Frequency of Ter119^+^ cells among live BM. Points represent individual mice, and lines represent mean. Groups were compared by one-way ANOVA with Tukey’s test; representative of two independent experiments. *, P < 0.05; **, P < 0.01; ***, P < 0.001; ****, P < 0.0001.

To explore the transcriptomic events occurring after erythroid progenitors are exposed to IL-33, we sorted pre–CFU-Es from healthy mice and cultured them for 6 h in vitro before performing bulk RNA-sequencing analysis (Fig. 6 A). Control and IL-33–exposed pre–CFU-Es clustered separately based on differential expression of 482 genes, of which 388 were up-regulated and 94 down-regulated (Fig. 6 B and Fig. S5 A). Among those genes down-regulated were several with known roles in erythroid differentiation, including vimentin (*Vim*) required for enucleation (Xue et al., 1997) and the enzyme coproporphyrinogen III oxidase (*Cpox*) required for Hgb synthesis (Taketani et al., 2001), whereas CD69, which inhibits erythroid differentiation (Huang et al., 2018), was up-regulated (Fig. 6 B and Fig. S5 B).

Among the genes strikingly up-regulated by IL-33 were many members of the NF-κB pathway (Fig. 6, B and C), which transmits IL-33 signals in other cell types (Pinto et al., 2018). Members of both canonical and noncanonical pathways were up-regulated (Fig. 6 D), and we confirmed that IL-33 increased nuclear translocation of the p65 subunit (encoded by *Rela*) in erythroid progenitors by approximately twofold using imaging flow cytometry (Fig. 6, E and F). To define its functional role, we inhibited the canonical NF-κB pathway using a NEMO domain–binding (NBD) peptide that inhibits IκB kinase γ (Fig. 6 G). In cultures of sorted pre–CFU-Es, inclusion of this peptide inhibited the effect of IL-33, alleviating its suppression of development of Ter119^+^ cells by ∼50% (Fig. 6 H). Collectively, these observations confirmed that activation of NF-κB signaling, which normally decreases with progressive erythroid maturation (Zhang et al., 1998), was required for the suppressive action of IL-33.

### IL-33 inhibits phosphorylation events downstream of the EPO-R, producing resistance to EPO-stimulated erythropoiesis

**Figure 7.**
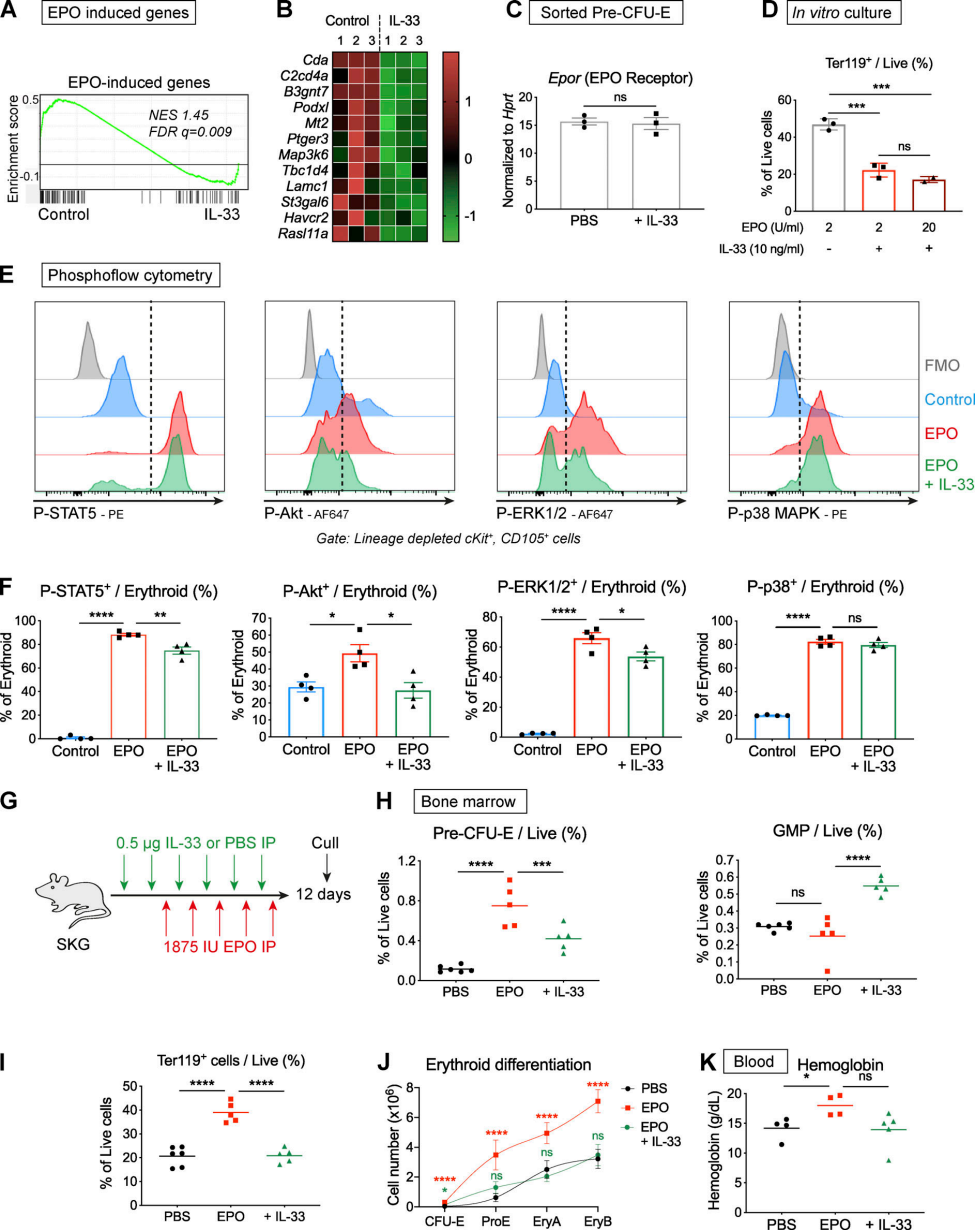
**IL-33 interferes with signal events downstream of the EPO receptor, curtailing EPO-accelerated erythropoiesis.** **(A)** Enrichment plot of GSEA in pre–CFU-Es using a set of genes up-regulated by EPO in CEPs. FDR q, false discovery rate q value; NES, normalized enrichment score. **(B)** Heatmap showing differential expression of selected genes in pre–CFU-Es with exposure to IL-33 that were induced by EPO in CEP, expressed as row-normalized z scores. Columns show paired replicates from *n* = three independent experiments, each with *n* = six mice. **(C)** Expression of *Epor* by RT-qPCR in pre–CFU-Es sorted by FACS from littermate SKG mice injected with PBS or IL-33 for 1 wk. Expression normalized to *Hprt* (mean and SEM of *n* = three independent experiments, *n* = two per group, unpaired *t* test). **(D)** Proportion of cells expressing Ter119 after culture of FACS-sorted pre–CFU-Es with EPO at 2 U/ml or 20 U/ml, ±IL-33 (mean and SD of technical triplicates, one-way ANOVA with Tukey’s test, representative of two independent experiments). **(E)** Representative images showing flow cytometric expression of indicated phosphorylated proteins in erythroid progenitors exposed to medium or IL-33 for 6 h then stimulated with EPO. Dashed lines indicate cutoff for positive cells. Representative of four independent experiments. **(F)** Proportion of erythroid progenitors expressing indicated phosphorylated proteins (mean of *n* = four independent experiments, *n* = three replicates per group for each experiment, with mean and SEM, one-way ANOVA with Tukey’s test). **(G)** Schematic diagram showing injection of littermate SKG mice with IL-33 or PBS, followed by EPO, according to indicated schedule before culling to generate data shown in H–K. **(H)** Frequencies of indicated progenitor populations in BM (points show individual mice with mean, one-way ANOVA with Tukey’s test, representative of two independent experiments). **(I)** Frequencies of Ter119^+^ erythroid cells in BM (points show individual mice with mean, one-way ANOVA with Tukey’s test, representative of two independent experiments). **(J)** Erythroid differentiation pathways (mean and SD of *n* = five mice per group, locally weighted scatterplot smoothing (LOWESS) spline, one-way ANOVA with groups compared with PBS group using Tukey’s test, representative of two independent experiments). **(K)** Blood Hgb concentration (points show individual mice with mean, one-way ANOVA with Tukey’s test, representative of two independent experiments). *, P < 0.05; **, P < 0.01; ***, P < 0.001; ****, P < 0.0001.

Analyzing the genes down-regulated by IL-33 in sorted pre–CFU-Es in more detail, we observed considerable overlap between these and a group of genes up-regulated in equivalent progenitors by injection of EPO in mice in a previous study (Fig. 7, A and B; Tusi et al., 2018). These genes were enriched in IL-33–naive pre–CFU-Es in our analysis (Fig. 7 A), leading us to hypothesize that IL-33 might interfere with the action of EPO in these cells. Exposure to IL-33 did not cause down-regulation of the *Epor* gene, encoding the EPO-R (Fig. 7 C), or loss of the protein from the cell surface (Fig. S5 C), nor did use of a 10-fold greater concentration of EPO rescue IL-33–induced inhibition in culture (Fig. 7 D). Collectively, this suggested any effect of IL-33 on EPO-R signaling would be downstream of the surface receptor itself.

Upon binding to EPO, the cell surface EPO-R dimerizes and causes activation of several intracellular signaling cascades, including JAK2-STAT5, PI3K-ERK1/2, p38 MAPK, and PI3K-Akt (Watowich, 2011). Of these, JAK2-STAT5 activates Bcl-xL to prevent apoptosis of erythroid progenitors (Silva et al., 1999), whereas Akt promotes differentiation (Ghaffari et al., 2006) by phosphorylating the essential erythroid transcription factor GATA1 (Zhao et al., 2006) and activating metabolic pathways via phosphorylation of the mTORC1 complex (Meng et al., 2018). To assess the effect of IL-33 on these pathways, we incubated lineage^neg^ BM preparations with IL-33 or medium and then stimulated them with EPO before assessing phosphorylation of critical molecules in CD105^+^ erythroid progenitors (i.e., CFU-Es and pre–CFU-Es) by phosphoflow cytometry. In control conditions, EPO activated all four pathways downstream of EPO-R but, with prior incubation with IL-33, there was consistent suppression of STAT5, ERK1/2, and, more strikingly, Akt phosphorylation in response to EPO (Fig. 7, E and F). Among the substrates of Akt is the mTORC1 complex, which phosphorylates S6 kinase and other targets to enhance protein translation and cellular metabolism (Meng et al., 2018). Activity of the mTORC1/S6 pathway is increased with progressive erythroid differentiation (Liu et al., 2017), and, in murine erythroid progenitors, we found increased phosphorylation of mTOR and S6 kinase with EPO stimulation (Fig. S5 D). However, phosphorylation of neither molecule was affected by IL-33 exposure (Fig. S5 D), indicating IL-33–induced suppression of Akt phosphorylation did not impair EPO-induced mTORCI/S6 kinase activation.

Inflammation has been implicated as a factor causing resistance to therapeutic administration of EPO in patients with chronic anemia (Macdougall and Cooper, 2002); we hypothesized that IL-33 could contribute to this effect owing to its impact on EPO-R signaling. We therefore injected EPO in mice to produce a model of accelerated erythropoiesis (Fig. 7 G), which occurs during severe anemia to increase production of RBCs. Whereas injection of EPO alone caused marked increases in the numbers of BM erythroid progenitors (Fig. 7 H), BM Ter119^+^ erythroid cells (Fig. 7 I), erythroid differentiation pathway (Fig. 7 J), and blood Hgb (Fig. 7 K), concurrent administration of IL-33 largely abrogated these responses. To understand the relevance of this finding in the setting of inflammation, we injected EPO, with or without curdlan, in WT mice (Fig. S5 E), finding that curdlan clearly suppressed the EPO-induced expansion of erythroid progenitors and Ter119^+^ cells in BM (Fig. S5, F and G). However, this effect was partially inhibited in IL-33^−/−^ mice, indicating IL-33 production contributed to the EPO resistance produced by curdlan (Fig. S5, F and G).

Collectively, these results show that IL-33 inhibits EPO-induced erythropoiesis in vitro and in vivo, which may be attributable to decreased activity in the Akt and, to a lesser extent, ERK1/2 and STAT5 signaling pathways. These observations provide a mechanism for the direct inhibition of differentiation of erythroid progenitors caused by IL-33 in the context of chronic inflammation.

## Discussion

**Figure 8.**
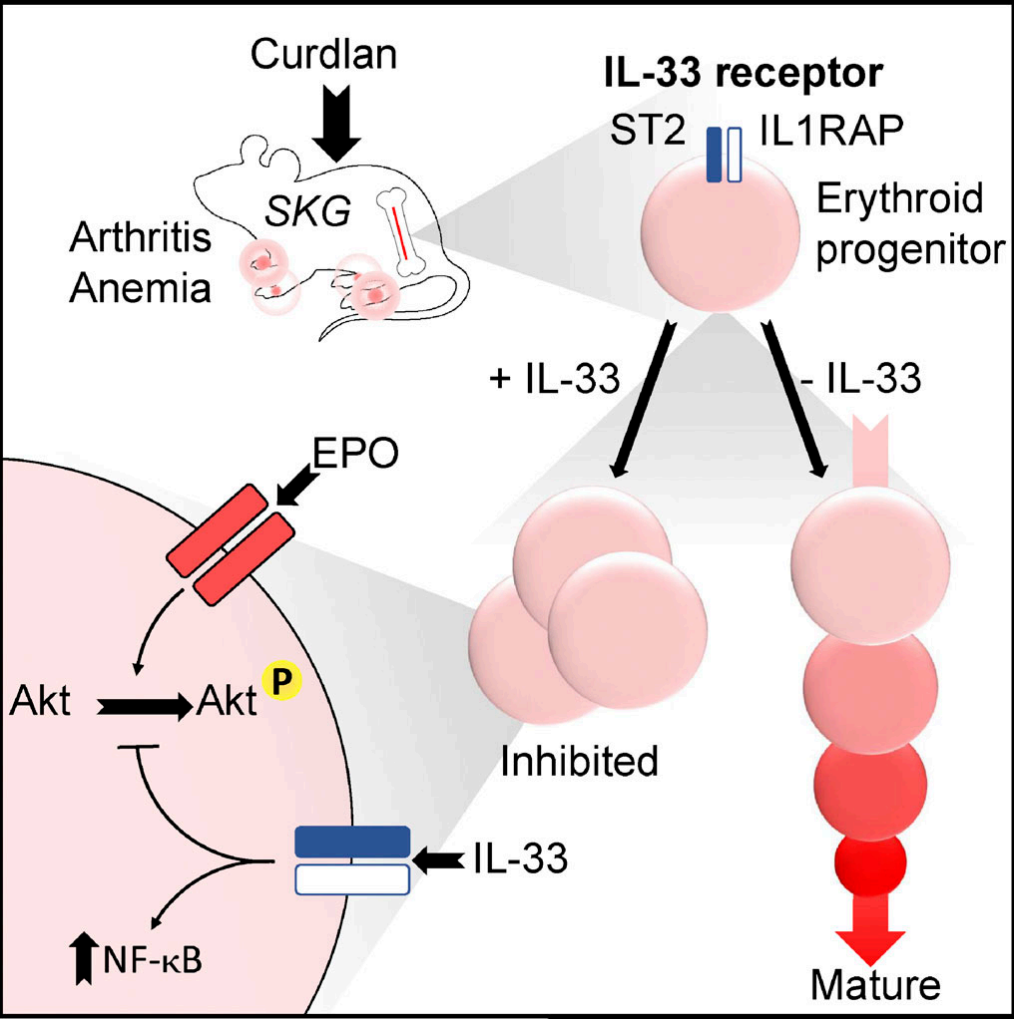
**IL-33 promotes AID by inhibiting differentiation of erythroid progenitors.** Erythroid progenitors preferentially express the receptor for IL-33, and exposure to IL-33 inhibits erythroid differentiation in vitro. This effect is attributable to increased NF-κB activation and is associated with decreased phosphorylation of Akt after EPO stimulation.

Our study reveals a previously unknown role for IL-33 in specific suppression of murine and human erythropoiesis in vitro and shows this suppression is necessary and sufficient to cause AID and EPO resistance in experimental models of chronic inflammation (Fig. 8). Mechanistically, we show these effects are dependent on NF-κB activation and associated with decreased responsiveness of signaling pathways downstream of EPO-R.

We found the effects of IL-33 in erythroid progenitors are dependent on NF-κB activation, and NF-κB is a common pathway used by IL-1 cytokine family members (Fields et al., 2019). However, in the context of erythropoiesis, this is important because activity of many NF-κB constituents, including p65, p50, and p52, decreases with progressive differentiation (Zhang et al., 1998). Studies of human and murine erythroid cell lines suggest high levels of NF-κB activity repress transcription at important loci required in differentiated cells, including the globin genes (Liu et al., 2003). Based on this and our own observation that NF-κB inhibition rescued cells from IL-33–mediated inhibition in vitro, we suggest IL-33 maintains inappropriately increased levels of NF-κB activity that prevent terminal differentiation of erythroid progenitors.

Building on this observation, we show that IL-33 suppresses EPO-induced Akt phosphorylation. This is important because EPO has a central and indispensable role in supporting erythroid cells during the final stages of differentiation, to the extent that EPO- and EPO-R–deficient mice suffer embryonic lethality (Wu et al., 1995). Interestingly, the onset of EPO dependence occurs shortly after formation of CFU-Es, coinciding with point at which IL-33 prevented further differentiation in our cultures. Among its pleiotropic effects on erythroid cells, EPO-induced Akt activation promotes differentiation because Akt phosphorylates and activates GATA1 (Zhao et al., 2006), providing a possible explanation for our observation that inhibition of Akt activity is associated with truncated differentiation. Interestingly, IL-33 had little or no effect on p38 MAPK, STAT5, or ERK1/2 phosphorylation, which appears to reconcile with the lack of effect of IL-33 on Ki67 and annexin V expression on pre–CFU-Es in vitro.

We confirmed IL-33 promoted myelopoiesis when administered alone (Regan-Komito et al., 2020; Talabot-Ayer et al., 2015) but was dispensable for curdlan-induced expansion of the GMP pool, suggesting it acts indirectly or alongside other cytokines to produce this effect. Teleologically, we suggest the simultaneous effects of IL-33 to permit myelopoiesis and suppress erythropoiesis could enable organisms to respond to transient damage or infection, taking advantage of the long circulating time of mature RBCs to provide a buffer against the development of anemia while BM output is diverted to meet other demands. During inflammatory diseases, this process would become detrimental if suppression of erythropoiesis were prolonged, resulting in AID as RBCs were lost but not replaced.

With curdlan injection, we show the concentration of IL-33 increased in BM plasma, an effect that could be attributable to local production in the BM niche or transport of IL-33 in the bloodstream from inflamed sites. We recently showed that development of SpA in SKG mice is associated with increased IL-33 production in the inflamed paws and small intestine (Regan-Komito et al., 2020), and others have found IL-33 is increased in the serum of patients with ankylosing spondylitis (Han et al., 2011; Li et al., 2013), the most common form of SpA in people. This suggests local production of IL-33 in the BM niche could be superseded by distant production in inflamed tissues during disease, which would reconcile with decreased expression of *Il33* in CD169^+^ macrophages in mice developing SpA.

Completion of further experiments for this study has been limited by the pandemic of SARS-CoV-2. Although curdlan injection in WT mice produced a similar hematopoietic phenotype to that seen in SKG mice with SpA, we would ideally have injected SKG mice with a neutralizing antibody against IL-33 as they developed SpA to assess the contribution of IL-33 to AID and EPO resistance in the context of inflammatory arthritis. Additionally, we would have investigated the kinetics of IL-33 production in more detail during disease by injecting citrine reporter mice with curdlan and by measuring the concentration of the soluble ST2 receptor, which sequesters IL-33 and curtails its activity (Liew et al., 2016), in serum and BM plasma as SpA developed in SKG mice.

Collectively, our results highlight IL-33 as a novel contributor to AID through its effects on differentiation of erythroid cells. Consequently, IL-33 may represent a therapeutic target for patients with AID or EPO resistance for amelioration of an important comorbidity of chronic inflammation.

## Materials and methods

### Mice and models of disease

All mice were bred and maintained in specific pathogen-free conditions at the University of Oxford. Experiments were conducted with appropriate licenses issued under the UK Animal (Scientific Procedures) Act 1986 and with approval from the University of Oxford animal welfare ethical review board. C57BL/6Nq-*p55*^−/−^ and C57BL/6Nq-*p75*^−/−^ mice were provided by the laboratory of Professor Richard Williams (University of Oxford), SKG mice were provided by Professor Shimon Sakaguchi (Osaka University, Suita, Japan), C57BL/6-*Il1rl1*^−/−^ mice were provided by Professor Padraic Fallon (Trinity College Dublin, Dublin, Ireland), and BALB/C-*Il33^cit/+^* mice were provided by Professor Andrew McKenzie (University of Cambridge, Cambridge, UK). The latter mice were backcrossed onto the C57BL/6 background until the progeny were at least 99.9% pure based on single-nucleotide polymorphism–based genotyping, and the mice were intercrossed to generate homozygous *Il33^cit/cit^* mice. WT C57BL/6, C57BL/6-*Il6*^−/−^, and C57BL/6-*Ubc^GFP^* mice were purchased from The Jackson Laboratory and bred at the University of Oxford.

SKG mice (BALB/C-*Zap70*^W163C^) are predisposed to development of autoimmune responses, owing to a spontaneous mutation in the gene encoding the ZAP-70 tyrosine kinase that forms part of the T cell receptor signaling complex. These mice are healthy under specific pathogen–free conditions but develop inflammatory arthritis, enteritis, uveitis, and dermatitis 4–5 wk after IP injection of 0.3 mg of the β-1,3-glucan curdlan, derived from *Alcaligenes faecalis* (Wako Chemicals). Mice of a single sex were used in each experiment.

After injection of curdlan (0.3 mg IP) in SKG mice, development of arthritis was assessed by weekly measurements of the paws and tarsi using calipers and using the following clinical score: 0 = no swelling or redness; 0.5 = swelling or redness of digits and mild swelling and/or redness of carpi or tarsi; 1 = mild swelling of carpal and tarsal joints; 2 = moderate swelling of carpal and tarsal joints; and 3 = substantial swelling of carpal and tarsal joints not inhibiting normal mobility, feeding, and drinking. Scores for forepaws and hindpaws were summated for each mouse. Changes in caliper measurements of the paws and tarsi were expressed as percentage changes compared with measurements made on the day of curdlan injection.

Where indicated, male C57BL/6 (WT) and C57BL/6-*Il33*^cit/cit^ (expressing a citrine reporter in place of the *Il33* gene, IL-33^−/−^) were also injected with the same dose of curdlan and culled after 1 wk.

Where indicated, male SKG mice were injected with PBS or curdlan, followed by IP injections of 0.5 µg recombinant murine IL-33 (BioLegend) according to one of the following schedules: (1) one injection every 2 d for a total of four injections, with mice culled the day after the last injection; or (2) one injection twice weekly for 4 wk. C57BL/6-*Il6*^−/−^, C57BL/6Nq-*p55*^−/−^, and *p75*^−/−^ mice were also injected with IL-33 or PBS according to schedule 1.

Where indicated, female SKG mice were injected with 0.5 µg IL-33 or PBS every 2 d for six injections. In some of these mice, EPO (BioLegend) was injected (1,875 U/injection, ∼75,000 U/kg, IP) every 2 d for five injections, starting after two injections of IL-33 had been administered. IL-33 and EPO were administered on alternate days thereafter until the mice were culled. Where indicated WT or IL-33^−/−^ mice were injected with PBS or curdlan (0.3 mg IP) and then EPO (1,875 U/injection every 2 d for four injections), culling the day after the last dose of EPO was injected.

### Isolation of cells from BM for analysis by flow cytometry

Femurs and tibiae were harvested into PBS supplemented with 0.1% BSA. BM cell suspensions were prepared by flushing the marrow from the central cavities of the femur and tibia using 5 ml PBS/0.1% BSA, 25G needles, and 1-ml syringes before breaking clumps by pipetting 10–20 times with a P1000 pipette and passing through a 70-µm filter.

### Isolation of cells from spleen for analysis by flow cytometry

The whole spleen was harvested into PBS/0.1% BSA. A cell suspension was prepared by crushing the spleen through a 70-µm filter using the barrel of a 5-ml syringe and 10 ml PBS/0.1%. The suspension was centrifuged, resuspended with 2 ml RBC lysing buffer (Sigma-Aldrich) for 2 min, and then washed, gross clumps were removed, and resuspended in PBS/0.1% BSA for staining.

### Isolation of cells from the soft tissue of the paw for analysis by flow cytometry

The skin was removed from one hind paw and the paw was cut into four pieces. Pieces of tissue were incubated in 1 ml RPMI 1640 supplemented with DNase I (0.1 mg/ml; Sigma-Aldrich) and Liberase (0.3 mg/ml; Roche) for 90 min at 37°C. Digested soft tissue from the paw joint was then passed through a 70-µm strainer to obtain a single-cell suspension, which was stained for flow cytometry.

### Flow cytometry

Single-cell suspensions of desired tissues were obtained as described above. 1–2 × 10^6^ cells were stained with fixable viability dye (Zombie Aqua or Zombie Green; BioLegend), unlabeled anti-CD16/32 (BioLegend) to block nonspecific staining (unless anti-CD16/32 was in the staining panel), and surface antibodies in PBS with 0.1% BSA for 30 min in the dark at 4°C. Cells were centrifuged and fixed for 15 min using fixation solution (BD Cytofix/Cytoperm buffer) at 4°C. Cells were centrifuged and resuspended in PBS with 0.1% BSA and 2 mM EDTA. The following anti-mouse antibodies were used (all from BioLegend, unless otherwise stated): anti-CD45 AF700 clone 30-F11, anti-CD11b BV510 or peridin-chlorophyll protein (PerCP)/Cy5.5 clone M1/70, anti-Ly6G BV421 clone 1A8, anti-CD3 PerCP/Cy5.5 clone 17A2, anti-CD4 PerCP/Cy5.5 clone GK1.5, anti-Gr1 PerCP/Cy5.5 clone RB6-8C5, anti-B220 PerCP/Cy5.5 clone RA3-6B2, anti-Ter119 allophycocyanin (APC) clone Ter119, anti-CD71 PE or FITC clone RI7217, anti-CD16/32 APC/Cy7 or PE/Cy7 BD clone 2.4G2, anti-CD150 BV605 clone TC15-12F12.2, anti-Conceptualization CD117 BV786 or PE BD clone 2B8, anti-CD41 PE/Cy7 clone MWReg30, anti-CD105 Pacific blue or APC clone MJ17/18, anti-Sca1 BV711 or Pacific blue clone D7, anti-IFNγR α chain biotin clone 2E2, anti-ST2 PE clone DIH9, anti-TNFRI/p55 PE clone 55R-170, anti-TNFRII/p75 AF647 AbD Serotec clone MCA351A648T, anti-GMCSFRa APC R&D Systems clone 698423, anti-CD169 APC clone 3D6.112, anti-F4/80 PE clone BM8, anti-Ly6C BV711 clone HK1.4, anti-Gr1 FITC eBioscience clone RB6-8C5, anti-CD16/32 APC clone 93, and anti-NF-κB p65 PE Santa Cruz Biotechnology clone F-6. The following anti-human antibodies were used (all from BioLegend): anti-CD3 biotin clone DKT3, anti-CD71 PE/Cy7 clone CY1G4, anti-CD19 biotin clone HIB19, anti-CD14 biotin clone 63D3, anti-CD235a APC clone HI264, anti-CD123 APC clone 6H6, and anti-CD34 FITC clone 561.

For Ki67 staining, cells were stained as above then fixed and permeabilized using the FoxP3/transcription factor staining buffer (eBioscience) set according to the instructions provided. Cells were then washed and stained for Ki67 (anti-Ki67 PE clone 16A8, 5 µl per sample) in 1× permeabilization buffer for 45 min in the dark at 4°C before washing and resuspending in PBS/0.1% BSA and 2 mM EDTA for acquisition.

For annexin V staining, cells were stained as above then resuspended in annexin V binding buffer (BioLegend) for 15 min in the dark at 4°C. Cells were washed and stained with PE-conjugated annexin V (5 µl per sample; BioLegend) for 45 min in the dark at 4°C, before washing and resuspending in binding buffer for acquisition.

Samples were acquired on an LSR II or LSR-Fortessa (BD) and analyzed using FlowJo Software.

### FACS

BM suspensions were prepared as described above, and lineage-positive cells were depleted using a lineage depletion kit (eBioscience mouse hematopoietic lineage biotin panel; ThermoFisher Scientific) according to the manufacturer’s instructions. Briefly, BM derived from the femurs and tibiae of three mice was incubated with biotinylated antibodies against CD3, B220, Gr1, and Ter119 in a volume of 1 ml PBS/0.1% BSA for 10 min at 4°C. Cells were then washed and incubated with 0.4 ml streptavidin bound to magnetic beads (Magnisort beads; ThermoFisher Scientific) in a volume of 2 ml PBS/0.1% BSA in a FACS tube for 10 min at room temperature. The volume was then made up to 4 ml, and the FACS tube was placed inside a magnet for 10 min at room temperature. The fraction of cells not bound to the magnet was poured out and stained for FACS as described above.

Pre–CFU-Es and CFU-Es were sorted by FACS using a FACS ARIA III (BD) with 70-µm nozzle. For culture, cells were sorted into 0.5 ml IMDM medium with 5% FCS in a 5-ml FACS tube. For RT quantitative PCR (RT-qPCR), cells were sorted into 350 µl RLT buffer with β-mercaptoethanol in 1.5 ml tubes. The following staining panel was used for sorting: Sca-1-Pacific blue, PE-CD117, APC-CD105, PerCP Cy5.5-CD11b and Streptavidin (BioLegend), APC Cy7-CD16/32, PE Cy7-CD41, BV605-CD150.

### ImageStream cytometry

BM suspensions were prepared using the lineage depletion kit, then resuspended in serum-free expansion medium (SFEM; Stem Cell Technologies). 1–2 × 10^6^ cells were plated in SFEM and rested for 15 min at 37°C and 5% CO_2_. Cells were then stimulated with medium or IL-33 (50 ng/ml) for 30 min and also stained for surface markers at the same time (CD105, Pacific blue; Gr1, FITC; CD16/32, APC; cKit, PE/Cy7; BioLegend), then centrifuged and fixed with warmed 4% formalin for 15 min. Cells were washed with PBS with 0.1% Triton X-100 (Sigma-Aldrich) and 3% FCS (Sigma Aldrich), then stained with NF-κB p65-PE (5 µl per sample in the same buffer; Santa Cruz Biotechnology) for 1 h at room temperature in the dark. Cells were centrifuged and washed, then stained with 7AAD (15 µg/ml in the same buffer; BioLegend) for 1 h at room temperature. Cells were washed and resuspended in PBS/0.1% BSA for acquisition.

Samples were acquired with an Amnis ImageStream XMarkII with the following settings: magnification 60 µm, flow rate slow. Translocation of the p65 probe was assessed in erythroid progenitors (cKit^+^, CD16/32^−^, CD105^+^) using the nuclear translocation wizard in IDEAS6.2 software (Amnis).

### Phosphoflow cytometry

Single-cell suspensions of BM were obtained and lineage-positive cells were depleted as described above. The resulting suspensions of lineage-depleted BM cells were plated at 0.5–1 × 10^6^ cells/well, resuspended in SFEM, with or without IL-33 (10 ng/ml) and incubated for 6 h at 37°C and 5% CO_2_. After this, cells were stained with surface antibodies and viability dye (all BioLegend unless otherwise stated: CD105-Pacific blue; FITC-CD71, PE/Cy7-CD16/32 [BD], AF700-CD11b, BV786-CD117; [BD]) and simultaneously stimulated with EPO (4 U/ml) for 20 min and then fixed with warmed BD Cytofix/Cytoperm solution for 12 min. Cells were centrifuged, washed with PBS/0.1% BSA, and permeabilized with Fixation buffer III (cooled to −20°C; BD) on ice for 30 min. Cells were then washed with PBS/0.1% BSA and stained with phospho antibodies (anti-STAT5 pY694 PE clone 47/Stat5(pY694), anti-Akt pS473 AF647 clone M89-61, anti-ERK1/2 pT202/pY204 AF647 clone 20A, anti-p38 MAPK pT180/pY182 PE clone 36/p38, anti-mTOR pS2448 PE clone MRRBY, and anti-pS6 pS235/pS236 PE clone N7-548, 5 µl/sample; all from BD) for 1 h at room temperature in the dark. Cells were then washed and acquired as described above. Erythroid progenitors were gated as CD11b^−^cKit^+^CD16/32^−^CD105^+^.

### Complete blood cell counts

Blood was collected from mice by cardiac puncture after death was confirmed. Blood (450 µl) was collected into a 1-ml syringe containing 50 µl 4% sodium citrate (Sigma-Aldrich) and then transferred to a plain 1.5-ml tube and kept at 4°C. Within 4 h, samples were gently mixed and analyzed using a Sysmex PX21 analyzer. Results were corrected for the dilution effect of the anticoagulant (corrected value = [measured value / 9] × 10).

### BM colony-forming assays

BM was prepared as described above and RBCs were lysed by mixing the cell pellet with 0.5 ml of RBC lysis buffer (Sigma Aldrich) and incubating for 2 min at room temperature. Cells were washed with PBS/0.1% BSA and counted. 5 × 10^4^ cells were resuspended in 100 µl of IMDM medium (Gibco) with 5% FCS and then mixed with 1 ml M3434 Methocult medium (Stem Cell Technologies). Suspensions were thoroughly mixed by vortexing and shaking before plating into 35-mm Petri dishes. Where indicated, BM cells were incubated with or without recombinant murine IL-33 (10 ng/ml) in liquid IMDM medium with 5% FCS for 6 h before mixing with M3434 Methocult that also contained IL-33 (10 ng/ml). Dishes were sealed with Micropore tape, placed inside a 10-cm Petri dish with a dish of sterile water, and incubated at 37°C and 5% CO_2_ for 10 d. Colonies were assessed and counted manually using an inverted microscope. Images were obtained at 10× magnification using an Olympus BX51 microscope.

### BM plasma

The BM of single femurs was flushed into 1.5-ml tubes using 250 µl of PBS/0.1% BSA, 1-ml syringes, and 25G needles. The suspension was vortexed briefly and then centrifuged at 13,000 *g* for 10 min. The supernatant was removed into a new tube and frozen at −80°C until assayed.

Concentrations of TNF-α and IL-33 were measured in BM plasma using ELISAs according to the manufacturer’s instructions (TNF, BioLegend Legend Max; IL-33, R&D Systems DuoSet) except that, for the IL-33 ELISA, samples and standards were incubated for 6 h with orbital shaking.

### Bulk culture of pre–CFU-Es

Pre–CFU-Es (defined as lineage^neg^, Sca1^neg^, cKit^+^, CD41^neg^, CD16/32^neg^, CD105^+^, CD150^hi^) sorted by FACS were resuspended in SFEM supplemented with recombinant murine SCF (100 ng/ml; BioLegend), recombinant human EPO (2 U/ml), and, where indicated, recombinant murine IL-33 (10 ng/ml) or TNF-α (10 ng/ml; BioLegend). All cultures were completed in triplicate, with 1,000–2,000 cells in 200 µl volume per well of a 96-well tissue culture plate incubated at 37°C and 5% CO_2_. After 2 d, 60 µl medium was removed and replaced with fresh medium. After 4 d, plates were centrifuged and stained for flow cytometry as described above. Counting beads (BioLegend) were used to measure the number of cells in each well.

For assessment of morphology, some cells were incubated for 4 d, washed twice with plain PBS, and then spun onto plain glass slides using a Shandon Cytospin centrifuge with the following settings: medium acceleration, 800 rpm, 5 min. The slides were stained with Shandon Kwik Diff solutions (ThermoFisher Scientific) according to the manufacturer’s instructions; images were obtained using an Olympus BX51 microscope at 40× magnification.

For assessment of enucleation by flow cytometry, some cells were incubated for 4 d and then incubated with Hoechst 33342 (20 µg/ml) in SFEM for 30 min at 37°C and 5% CO_2_. The cells were centrifuged and stained with anti-Ter119-APC and CD71-PE (BioLegend) before acquisition as described above.

For assessment of colony formation, 1,000 sorted pre–CFU-Es were resuspended in SFEM with SCF and EPO (as described above), with or without IL-33 (10 ng/ml) and incubated for 6 h at 37°C and 5% CO_2_. Cells (in a volume of 300 µl) were then mixed with 0.8 ml M3236 Methocult (Stem Cell Technologies) supplemented with SCF (100 ng/ml) and EPO (2 U/ml), with or without IL-33 (10 ng/ml), and plated onto 35-mm Petri dishes. Dishes were sealed with Micropore tape and incubated for 48 h. The number of CFU-E colonies, identified as 8–32-cell clusters forming a distinct colony, was counted manually for each dish.

For assessment of the direct/indirect effects of IL-33, pre–CFU-Es were sorted as above from C57BL/6-*Il1rl1*^−/−^ (ST2 knockout) and C57BL/6-*Ubc*^GFP^ mice (WT, expressing GFP under the control of the ubiquitin c gene). These cells were plated individually (2,000 cells per well) or mixed (1,000 cells of each type per well) and incubated and assessed as described above.

For assessment of the effect of NF-κB, NBD peptide was added (10 µM; Novus Biologicals) where indicated and left in the medium for 4 d. For assessment of the effect of a higher dose of EPO, EPO was added to the culture at a final concentration of 20 U/ml for 4 d.

### Single-cell culture of pre–CFU-E

BM suspensions were prepared as described above. Single pre–CFU-Es were sorted by FACS into wells of a 96-well plate, each containing 80 µl SFEM medium supplemented with SCF (100 ng/ml), EPO (2 U/ml), and dexamethasone (10 µM; Sigma-Aldrich), with or without IL-33 (10 ng/ml). Dexamethasone was added for single-cell cultures to increase proliferation to obtain a reliable readout. The outside row/column of the plate was filled with sterile water. Plates were incubated at 37°C and 5% CO_2_ for 10 d. At this time, 50 µl of medium was removed and replaced with 50 µl of PBS/0.1% BSA with viability dye (Zombie Aqua; BioLegend) and surface antibodies (CD71-PE, Ter119-APC). The plate was incubated at 4°C in the dark for 1 h, and then 50 µl was aspirated from each well and replaced with 150 µl PBS/0.1% BSA for acquisition by flow cytometry. The number of cells in each well was quantified using counting beads.

### Isolation and culture of human erythroid progenitors

Anonymized human leukoreduction cones were obtained from the NHS Blood and Transfusion Service, University of Oxford. Donors gave consent for the use of samples for research purposes. The cones were drained into 50-ml tubes and diluted with PBS to a final volume of 50 ml. From this, 25 ml was layered onto 15 ml Lymphoprep solution (Stem Cell Technologies) and centrifuged as follows: 600 *g*, 20 min, room temperature, acceleration 7, deceleration 0. The leukocyte layer was harvested with a P1000 pipette and washed three times with PBS.

From this suspension enriched for human PBMCs, 50 × 10^6^ cells were removed and mixed with 3 µg biotinylated antibodies against CD3, CD19, and CD14 (all BioLegend) in 1 ml PBS/0.1% BSA for 10 min at 4°C. Cells were washed and mixed with 0.4 ml streptavidin conjugated with magnetic beads in a final volume of 2 ml in a FACS tube and incubated at room temperature for 10 min. The volume was then adjusted to 4 ml, and the FACS tube was placed inside a magnet for 10 min. After this, the supernatant was poured into a fresh tube and stained with the following panel: FITC-CD34, APC-CD123, PE/Cy7-CD71, PerCP/Cy5.5-Streptavidin, and 7AAD. Erythroid progenitors were sorted as live, lineage^neg^ (i.e., PerCP/Cy5.5^neg^), CD34^+^, CD123^−^, CD71^+^ by FACS as described above.

Sorted cells were resuspended in SFEM with recombinant human SCF (100 ng/ml; Peprotech), recombinant human EPO (2 U/ml; BioLegend), with or without recombinant human IL-33 (10 ng/ml; Peprotech). Cultures were performed in triplicate, with 2,000 cells in 200-µl volume per well in a 96-well tissue culture plate. Every 2 d, 60 µl medium was removed and replaced with fresh medium. After 8–9 d, plates were centrifuged and cells were prepared for flow cytometry as described above. CD235a (glycophorin A) was used as a marker of mature erythroid cells in human cultures because there is no known equivalent to the Ter119 antigen.

### RT-qPCR

To sort cells for RT-qPCR, BM was prepared as described above under Flow cytometry. For stromal populations, femurs and tibiae were dissected, cleaned, and then crushed in a Petri dish. The bone fragments were transferred to wells of a 48-well plate with 1 ml of Hanks' buffered saline solution with DNase I (15 µg/ml; Sigma-Aldrich) and collagenase I (300 U/ml; Sigma-Aldrich). The plate was incubated at 37°C with shaking at 100 rpm for 1 h. After this, bone fragments were washed and, with the digestion solution, transferred to a new tube through a 70-µm filter.

For assessment of BM IL-33 production by RT-qPCR, the following populations were defined by FACS: mesenchymal stromal/stem cells: CD45^−^, Ter119^−^, CD31^−^, CD51^+^, PDGFRα^+^, Sca1^−^; endothelium: CD45^−^, Ter119^−^, CD31^+^; osteoblastic lineage cells: CD45^−^, Ter119^−^, CD31^−^, CD51^+^; erythroblastic island macrophages (CD169^+^ macrophages): CD45^+^, Gr1^−^, F4/80^+^, SSC^lo^, CD169^+^; neutrophils: CD45^+^, CD11b^+^, Ly6G^+^; and monocytes (Ly6C^+^ monocytes): CD45^+^, CD11b^+^, Ly6G^−^, Ly6C^+^

Progenitors or BM cell populations were sorted by FACS directly into RLT buffer with β mercaptoethanol, vortexed briefly, and frozen at −80°C until processed. RNA was extracted using the RNeasy Micro Kit (Qiagen), and 50 ng cDNA was synthesized using the High-Capacity Reverse Transcription kit (Affymetrix eBioscience). RT-qPCR was performed using TaqMan primers/probes (Hba-a1 Mm02580841_g1, Hbb-b1 Mm01611268_g1, Alas2 Mm00802083_m1, Epb41 Mm01187466_m1, Gypa Mm00494848_m1, Epor Mm00833882_m1, Aqp1 Mm01326466_m1, Il33 Mm00505403_m1, and Il1b Mm00434228_m1; ThermoFisher Scientific) and PrecisionFast Mastermix (PrimerDesign) on a ViiA7 384-well real-time PCR system. All expression levels were normalized to an internal reference gene (*Hprt*) and calculated as 2^−(threshold cycle [CT]^ *^Hprt^*^− CT gene)^. Real-time qPCR reactions were performed in duplicate.

### Immunofluorescence

BM was prepared as described above, and CFU-Es were sorted by FACS. Cells were incubated in SFEM supplemented with EPO and SCF as described above at 9 × 10^4^ cells per well, with or without IL-33 (10 ng/ml) for 24 h. The plate was centrifuged and cells were fixed in 4% formalin for 20 min at room temperature. Cells were washed once with PBS/0.1% BSA and then stained with goat anti-mouse EPO-R antibody (15 µg/ml; R&D Systems) for 2 h at room temperature. Cells were then washed once and blocked with rabbit serum before staining with rabbit anti-goat IgG secondary AF555 antibody (1:500; Invitrogen) for 45 min at room temperature. Cells were washed once and then stained with Hoechst 33342 (Life Technologies) for 20 min. Cells were resuspended in 30 µl PBS/0.1% BSA, of which 10 µl was transferred to a plain glass slide and covered with a 1 cm diameter glass coverslip. After 10 min to allow cells to rest on the slide, cells were imaged using an Olympus BX51 microscope.

### RNA sequencing

Pre–CFU-Es were sorted by FACS and resuspended in SFEM with SCF and EPO as described above. Cells were incubated with or without IL-33 (10 ng/ml) for 6 h at 37°C and 5% CO_2_. Plates were then centrifuged, supernatants discarded, and cells transferred to 1.5-ml tubes with 350 µl RLT buffer with β-mercaptoethanol. RNA was extracted using the RNEasy Micro kit (Qiagen), according to the manufacturer’s instructions.

### RNA-sequencing analysis

RNA-sequencing data were analyzed using pipeline_scrnaseq.py (https://github.com/sansomlab/scseq/blob/master/pipelines/). Data quality was assessed using pipeline_readqc.py (https://github.com/cgat-developers/cgat-flow/). Sequence reads were aligned to the mouse genome with Hisat2 (version 2.1.0; Kim et al., 2015) using a “genome” index built from the GRCm38 release of the mouse genome and known splice sites extracted from Ensembl version 91 annotations (using the hisat2_extract_splice_sites.py tool). A two-pass mapping strategy was used to discover novel splice sites (with the additional parameters –dta and–score-min L,0.0,−0.2). The average alignment rate was 95.2% (as assessed with Picard Tools v2.10.9; https://github.com/broadinstitute/picard). Mapped reads were counted using featureCounts (Subread version 1.6.3; Ensembl version 91 annotations; with default parameters; Liao et al., 2014). Salmon v0.9.1 was used to calculate transcript per million values (Patro et al., 2017) using a quasi-index (built with Ensembl version 91 annotations and k = 31) and guanidine/cytosine (gc) bias correction (parameter “–gcBias”). Differential expression analysis of IL-33–exposed pre–CFU-E versus control samples was performed using DESeq2 (v1.24.0; Love et al., 2014). Raw data have been deposited in GEO (accession no. GSE141480).

### Gene set enrichment analysis (GSEA)

GSEA was performed using fgsea R-package (version 1.10.1) for Kyoto Encyclopedia of Genes and Genomes (KEGG) pathways (using for ranking the Wald statistic from DESeq2; Fig. 5 C; Sergushichev, 2016 *Preprint*). Enrichment of members of the NF-κB pathways and genes up-regulated by EPO in committed erythroid progenitors (CEPs) were assessed in RNA-sequencing results using GSEA software, version 4.0.2. Genes up-regulated in CEP by EPO were extracted from Supplementary Table 5 in Tusi et al. (2018). NF-κB pathway gene sets were obtained from the Pathway Interaction Database available through the GSEA software.

### Analysis of Haemosphere data

Results of RNA sequencing from murine hematopoietic progenitors were obtained from the online Haemosphere database, reporting the results of the Haemopedia sequencing project (Choi et al., 2019). Specifically, gene counts (expressed as transcript per million) were downloaded for CFU-E and GMP. Differential expression of genes between these cell types was evaluated using DEseq2 (v1.24.0) and EnchancedVolcano packages in R, version 3.6.1.

### Statistical analysis

Unless otherwise stated, all analysis was completed using proprietary software (GraphPad Prism). Groups were compared using unpaired *t* tests, Mann-Whitney *U* tests, one-way ANOVA, or two-way ANOVA depending on the number of groups and nature of comparisons. If significant differences were detected by ANOVA, post hoc Tukey tests (one way) or Sidak’s multiple comparison tests (two way) were completed. Correlations were assessed using linear regression analysis.

### Online supplemental material

Fig. S1 presents additional data on the hematopoietic changes observed in SKG mice developing SpA and shows similar data from WT C57BL/6 mice injected with curdlan. Fig. S2 provides additional data on the expression of the IL-33 receptor ST2 on lineage-committed and MPPs. Fig. S3 provides additional data on proliferation and apoptosis markers for cultured erythroid cells. Fig. S4 provides additional data on the effect of IL-33 on hematopoiesis in mice lacking IL-6 or the receptors for TNF. Fig. S5 provides additional data on the expression of the EPO-R in cells exposed to IL-33 and on the dependence of curdlan on IL-33 to suppress EPO-accelerated erythropoiesis in vivo.
